# Efficacy and safety of cardioprotective drugs in chemotherapy-induced cardiotoxicity: an updated systematic review & network meta-analysis

**DOI:** 10.1186/s40959-023-00159-0

**Published:** 2023-02-18

**Authors:** Ali Mir, Yasra Badi, Seif Bugazia, Anas Zakarya Nourelden, Ahmed Hashem Fathallah, Khaled Mohamed Ragab, Mohammed Alsillak, Sarah Makram Elsayed, Abdulrahman Ibrahim Hagrass, Sawyer Bawek, Mohamad Kalot, Zachary L. Brumberger

**Affiliations:** 1 Department of Internal Medicine, Jacobs School of Medicine and Biomedical Sciences, University at Buffalo, Buffalo, NY USA; 2 All Saints University School of Medicine, Roseau, Dominica; 3 Henry Ford Macomb Hospital, Clinton Township, Macomb County, MI USA; 4 Faculty of Medicine, Al-Azhar University, Cairo, Egypt; 5 Faculty of Medicine, Minia University, Minia, Egypt; 6 Woodhull Medical and Mental Health Center Program, Brooklyn, NY USA; 7 Faculty of Medicine, October 6 University, Giza, Egypt; 8 Department of Medicine, Division of Cardiology, Jacobs School of Medicine and Biomedical Sciences, University at Buffalo, Buffalo, NY USA

**Keywords:** Cardiotoxicity, Cancer, Chemotherapy, Cardioprotective, Network meta-analysis

## Abstract

**Background:**

Cancer patients receiving chemotherapy have an increased risk of cardiovascular complications. This limits the widespread use of lifesaving therapies, often necessitating alternate lower efficacy regimens, or precluding chemotherapy entirely. Prior studies have suggested that using common cardioprotective agents may attenuate chemotherapy-induced cardiotoxicity. However, small sample sizes and conflicting outcomes have limited the clinical significance of these results.

**Hypothesis:**

A comprehensive network meta-analysis using updated and high-quality data can provide more conclusive information to assess which drug or drug class has the most significant effect in the management of chemotherapy-induced cardiotoxicity.

**Methods:**

We performed a literature search for randomized controlled trials (RCTs) investigating the effects of cardioprotective agents in patients with chemotherapy-induced cardiotoxicity. We used established analytical tools (netmeta package in RStudio) and data extraction formats to analyze the outcome data. To obviate systematic bias in the selection and interpretation of RCTs, we employed the validated Cochrane risk-of-bias tools. Agents included were statins, aldosterone receptor antagonists (MRAs), ACEIs, ARBs, and beta-blockers. Outcomes examined were improvement in clinical and laboratory parameters of cardiac function including a decreased reduction in left ventricular ejection fraction (LVEF), clinical HF, troponin-I, and B-natriuretic peptide levels.

**Results:**

Our study included 33 RCTs including a total of 3,285 patients. Compared to control groups, spironolactone therapy was associated with the greatest LVEF improvement (Mean difference (MD) = 12.80, [7.90; 17.70]), followed by enalapril (MD = 7.62, [5.31; 9.94]), nebivolol (MD = 7.30, [2.39; 12.21]), and statins (MD = 6.72, [3.58; 9.85]). Spironolactone was also associated with a significant reduction in troponin elevation (MD =  − 0.01, [− 0.02; − 0.01]). Enalapril demonstrated the greatest BNP reduction (MD =  − 49.00, [− 68.89; − 29.11]), which was followed by spironolactone (MD =  − 16.00, [− 23.9; − 8.10]). Additionally, patients on enalapril had the lowest risk of developing clinical HF compared to the control population (RR = 0.05, [0.00; 0.75]).

**Conclusion:**

Our analysis reaffirmed that statins, MRAs, ACEIs, and beta-blockers can significantly attenuate chemotherapy-induced cardiotoxicity, while ARBs showed no significant effects. Spironolactone showed the most robust improvement of LVEF, which best supports its use among this population. Our analysis warrants future clinical studies examining the cardioprotective effects of cardiac remodeling therapy in cancer patients treated with chemotherapeutic agents.

**Supplementary Information:**

The online version contains supplementary material available at 10.1186/s40959-023-00159-0.

## Introduction

The incidence of cancer is increasing worldwide and is a leading cause of death in both developed and developing countries. In 2020, the global cancer burden was estimated at an annual 19.3 million new cases and 10.0 million deaths. With increasing treatment, the cardiotoxic effects of chemotherapy have become more evident.

Treatment with trastuzumab has been noted to cause a significant LVEF decline in 7.1% to 18.6% of patients [1], and concomitant treatment with trastuzumab, an anthracycline, and cyclophosphamide has been noted to cause cardiac dysfunction in up to 27% of patients being treated for HER2 + metastatic breast cancer [2]. Cancer therapy-related cardiac dysfunction (CTRCD) is broadly defined as a decrease in LVEF of at least 10% to < 50% [3].

Anthracyclines are cytostatic chemotherapeutic agents that form the basis of treatment for many solid and hematological malignancies [4]. Anthracycline therapy is associated with an increased risk of developing heart failure with significant mortality and morbidity [5].

Cardiotoxicity is associated with an increased risk of morbidity and mortality in individuals with or without significant cardiovascular history [6]. Cardiotoxicity may range from subclinical to more overt conditions including irreversible cardiac failure and death [7] and can develop during the acute, early (< 1 year), or late (> 1 year) stages in the course of cancer treatment. Acute toxicities occur during or shortly after exposure to a chemotherapeutic agent, with symptoms of dyspnoea and chest tightness consistent with myopericarditis. Early toxicities occur within months of chemotherapy and typically manifest as acute onset heart failure with a reduced LVEF. Late toxicity occurs many years after treatment, as late-onset cardiomyopathy [8]. Approximately 15% of adult patients with a diagnosis of cardiomyopathy were treated for cancer during their childhood or adolescence [9].

Existing literature suggests that widely available cardiac medicines may lessen the effects of cardiotoxicity when used as a cardio-prophylactic therapy. Spironolactone has been found to have a cardioprotective effect in anthracycline-induced cardiotoxicity by preserving cardiac systolic and diastolic function and exerting an antioxidant effect through blockade of the RAAS, which normally facilitates cardiac oxidative damage such as that induced by anthracyclines [10]. Angiotensin-converting enzyme inhibitors (ACEi) and angiotensin receptor blockers (ARB) similarly act on the RAAS, suppressing the conversion of angiotensin I to angiotensin II and antagonizing the action of angiotensin-II, respectively.

Cardioselective beta-blockers (timolol, metoprolol, propranolol, bisoprolol, and carvedilol) have beneficial prognostic effects in patients with cardiac disease and dysfunction previous research on the benefit of beta blockers and ACEi in anthracycline and trastuzumab-induced cardiotoxicity has been equivocal. Beta-blockers have demonstrated a more favorable cardioprotective effect against anthracyclines and trastuzumab than ACEi/ARB. Further data on the combination of ACEi/ARB and B-blocker therapy in solid tumors is required to examine whether the beneficial additive effect seen in congestive heart failure can be replicated in CTRCD [11].

Previous studies and systemic reviews have examined the role of commonly used cardioprotective drugs in CTRCD. However, most of these studies have lacked the power to make definitive statements regarding the best agent(s) to use. This updated network meta-analysis aims to examine updated, high-quality and pooled data to better define the role of cardioprotective medication in CTRCD.

## Methods

### Search strategy and data collection

We searched PubMed, Cochrane library, Scopus, and Web of Science for matched records from inception until January 2022. The complete search can be found in Supplement 1. The prespecified protocol for this review is registered with PROSPERO (CRD42022366091). EndNote was used to aggregate the obtained records and look for any duplicates. We performed this NMA as recommended by “the Cochrane handbook” [12] and reported it using the NMA preferred reporting items [13, 14].

### Selection criteria

We included studies that met the following requirements:

(1) Population: patients with chemotherapy-induced cardiotoxicity; (2) Intervention: cardioprotective agents; beta-blockers, statins, ACE inhibitors, ARBs, aldosterone antagonists as well as individual drugs belonging to those families; (3) Comparator: cardioprotective agents or placebo; (4) Outcomes: LVEF (was measured by one of these methods; echocardiography, muga, MRI, or diastolic function), symptomatic heart failure (HF), BNP, and Troponin; (5) Study design: we restricted our search strategy to include RCTs only.

### Screening and data extraction

Independent reviewers conducted title and abstract screening and full-text review in duplicate to identify eligible studies. Two reviewers completed data extraction independently and in duplicate and data were verified by a third reviewer (A.Z.N). All disagreements were resolved with the group consensus.

We extracted the following information:

1. Characteristics of the enrolled population at the baseline and summary of the eligible trials including study ID (last name of first author/publication year), study arms, region, mean age, percentage of females, cancer type, type of chemotherapy, an accumulative dose of chemotherapy, left ventricular ejection fraction (LVEF), NT-proBNP, and troponin, inclusion and exclusion criteria of eligible studies, time of follow-up, primary endpoints, and conclusions
2. Outcomes: LVEF, symptomatic HF, BNP, and troponin
3. Domains of Cochrane random risk of a bias assessment tool for RCTs

### Quality assessment

We used the Cochrane risk-of-bias tool to assess the quality of the eligible studies [12]. It included the following domains: sequence generated randomly, concealed allocation (blinded participants and personnel), blinded assessors of outcomes, incomplete data, selective reporting, and others. Two authors independently judged each domain, with a third author resolving conflicts.

### Statistical analysis

To conduct this frequentist NMA, we used the netmeta package, which was available through RStudio. We pooled the data as mean difference (MD) for continuous outcomes and risk ratio (RR) for dichotomous outcomes with 95% CI (Confidence Interval) for each outcome. We examined and quantified considerable heterogeneity using the Chi-squared (Q2) and I-squared tests, respectively. We created forest plots for each of the pre-specified outcomes. We defined significant heterogeneity as a Breslow-Day Test I2 > 50% or *P*-value < 0.1. The random effect model was applied to resolve significant heterogeneity. All comparisons of estimated values acquired by our NMA were organized into a league table.

## Results

### Literature search

**Fig. 1:**
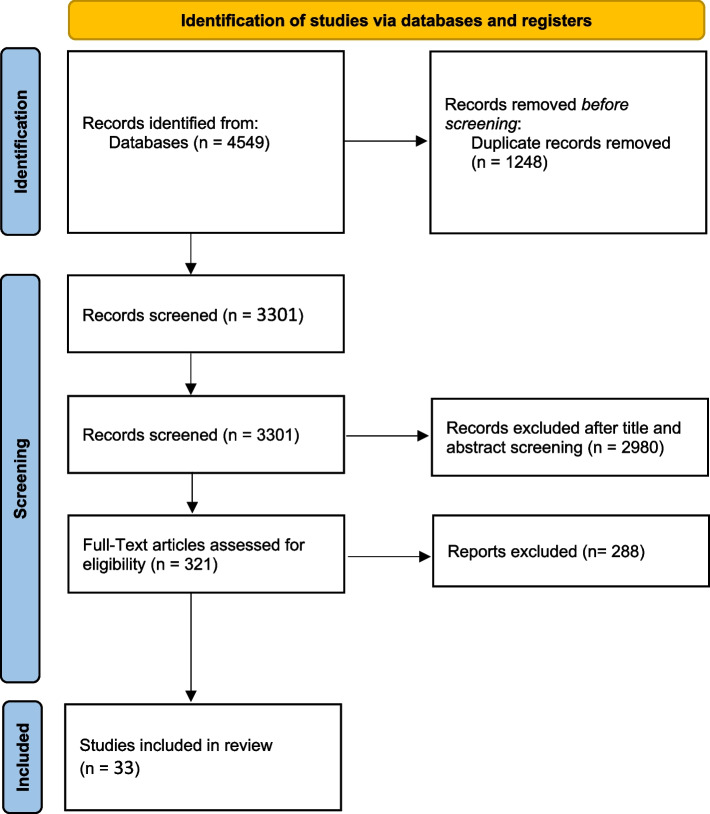
PRISMA Flow Diagram

The initial total records from our search on the four electronic databases were 4549. The total records then became 3301, following the removal of 1248 duplicates. After screening the titles and abstracts, the number of studies suitable for full-text screening was 321. Finally, 33 studies were included [10, 15–46]. Figure 1 shows a PRISMA Study flow diagram for the included studies.

### Summary of included studies

The total number of included patients from 33 studies was 3285. The included studies were conducted in the following countries; Brazil, Canada, Germany, Greece, India, Indonesia, Iran, Italy, Norway, Poland, Romania, Spain, Turkey, and the USA; and the most common country of origin was Iran, denoting eight studies [16, 31, 35–37, 41, 43, 46] However, none of the included studies were multinational. The study arms included candesartan + metoprolol, candesartan + placebo, metoprolol + placebo, placebo + placebo, carvedilol, placebo, enalapril, metoprolol, atorvastatin, enalapril + carvedilol, control, nebivolol, perindopril, telmisartan, spironolactone, statin, non-statin, candesartan, perindopril, bisoprolol, lisinopril and bisoprolol, ramipril, rosuvastatin, lisinopril, eplerenone, and ACEI. The most commonly reported outcome was the LVEF. We report all study characteristics in Table 1.

**Table 1:** Summary of included studies

Study ID	Region	Study arms, sample size	Dose	Other medications (Drug, number of patients)	Inclusion criteria	Outcomes	Conclusion
Gulati 2016 [28]	Norway	candestran + metoprolol, 30	32 mg + 100 mg	Trastuzumab, taxons	1-Women aged 18–70 years 2-Eastern Cooperative Oncology Group (ECOG) performance status 0–1 3-Serum creatinine < 1.6 mg/dL or estimated glomerular filtration rate (eGFR) ≥ 60 ml/min/1.73 m2 4-Systolic blood pressure ≥ 110 mmHg and < 170 mmHg 5-Left ventricular ejection fraction ≥ 50%	1-Change in LVEF by cardiac magnetic resonance imaging	"In patients treated for early breast cancer with adjuvant anthracycline-containing regimens with or without trastuzumab and radiation, concomitant treatment with candesartan provides protection against an early decline in global left ventricular function."
		candestran + placebo, 32	32 mg				
		metoprolol + placebo, 32	100 mg				
		placebo + placebo, 32					
Gupta 2017 [29]	India	enalapril, 44	of 0.1 mg/kg/day once a day from the first day of chemotherapy for 6 months	doxorubicin and/or daunorubicin	1- Patients aged more than 16 years at the time of diagnosis 2- Confirmed diagnosis of acute lymphoblastic leukemia/lymphoma (Hodgkin and non-Hodgkin lymphoma) 3-Estimated cumulative anthracycline dose ≥ 200 mg/m2	1-Measured decrease in LVEF (≥ 20%)	"Enalapril has a role in reducing cardiac toxicity after anthracycline administration."
		placebo, 40					
Kalay 2006 [33]	Turkey	carvedilol, 25	12.5 mg/day	adriamycin or epirubicin	1-Patients diagnosed with malignancy and planned ANT therapy (adriamycin or epirubicin) 2- Who have not received previous chemo or radiotherapy 3- Free of CHF and valvular disease	1-Left ventricular ejection fraction 2- Echocardiographic parameters	"Prophylactic use of carvedilol in patients receiving ANT may protect both systolic and diastolic functions of the left ventricle."
		placebo, 25	NA				
Cardinale 2006 [20]	USA	Enalapril, 56	an initial dose of 2.5 mg once daily and was increased gradually through 3 steps to 20 mg once daily (5, 10, and 20 mg, respectively)	NA	1-Cancer patients undergoing HDC 2-All patients with an early TnI value of 0.07 ng/Ml	1-Left ventricular ejection fraction 2-Echocardiographic parameters	"In high-risk, HDC-treated patients, defined by an increased troponin I value, early treatment with enalapril seems to prevent the development of late cardiotoxicity."
		placebo, 58					
Georgakopoulos 2010 [26]	USA	metoprolol, 42			1-The diagnosis of de novo DLBCL 2- Treated with R-CHOP with or without consolidation radiation therapy at the discretion of the attending physician 3- And be followed at Mayo Clinic Rochester	1-LVEF	“metoprolol and enalapril do not reduce the risk of cardiotoxicity in patients treated with doxorubicin.”
		enalapril, 43	NA	NA			
		placebo, 40					
Acar 2011 [15]	USA	atorvastatin, 20	40 mg	NA	1-Mean age of 53 15 years 2- Who was undergoing ANT chemotherapy was enrolled	1-Establishment of impairment in LV systolic functions defined as an ejection fraction (EF) of 50%	"prophylactic use of atorvastatin could be effective in the maintenance of LVEF in patients treated with ANT."
		placebo, 20	NA	NA			
Salehi 2011 [41]	Iran	placebo, 22		NA	1- Patients with a diagnosis of breast malignancies and lymphoma 2- Who was under treatment with anthracyclines 3- Those who were referred to Shahid Ghazi Clinic entered the study	1-LVEF	"Carvedilol at a daily dose of 12.5 mg has a protective effect against diastolic disorder and at a daily dose of 25 mg has a protective effect against both systolic and diastolic disorders."
		carvidelol, 22	12.5 mg	NA			
		carvidelol, 22	25 mg	NA			
Bosch 2013 [19]	Spain	Enalapril + carvedilol,45	Enalapril initial dose was 2.5 mg daiy hen increased gradulally every 3 o 6 days to 5 and 10 mg daily. Carvediolol's initial dose was 6.25 mg twice daily and gradually increased to 12.5 and 25 mg every 3 to 6 days	NA	1-Adult patients from 18 to 70 year-old 2-LVEF ≥ 50%	1-Absolute change from baseline LVEF 2-Serious adverse events 3-Death	" The combined treatment with enalapril and carvedilol may prevent LVSD in patients with malignant hemopathies treated with intensive chemotherapy. The clinical relevance of this strategy should be confirmed in larger future studies. relevance of this strategy for prevention of chemotherapy-induced cardiac damage should be confirmed in larger future studies."
		control,45		NA			
Kaya 2013 [34]	Turkey	nebivolol, 27	5 mg/day	adriamycin/epirubicin, cyclophosphamide, 5-Fluorouracil (5-FU) and docetaxel (DCT)-containing chemotherapy regimes	1-Female patients admitted from October 2007 to September 2008 to the medical oncology department of the Erciyes University Medical School for breast cancer	1-Left ventricular (LV) end-systolic and end-diastolic diameters	"Prophylactic use of nebivolol treatment may protect the myocardium against anthracycline-induced cardiotoxicity in breast cancer patients."
		placebo, 18					
Radulescu 2013 [39]	Romania	perindopril, 68	10 mg	epirubicin	patients admitted between 2007 to 2012 who had not undergone prior chemotherapies before admission	1-Left ventricular ejection fraction (EF)	"In the present echo-Doppler study we documented a preserved left ventricular systolic performance in patients with various malignancies treated with epirubicin plus perindopril. Although co-treatment with ACEI prevented the alteration of systolic performance, it failed to prevent the deterioration of the left ventricular diastolic performance impairment due to poor left ventricular compliance"
		Control,68	NA				
Dessì 2013 [23]	Italy	telmisartan, 25	40 mg	epirubicin	1-Patients aged 18–70 2- Blood pressure within the normal range (80/ 120) 3-Echocardiographic LVEF value ≥ 55% 4-SR value in the normal range (range: 1.7–2.1 cm/sec) 5- Eastern Cooperative Oncology Group (ECOG) performance status 6-Score of 0–2 (Oken, et al. 1982) 7- Normal hepatic and renal function (bilirubin ≤ 1.5 mg/dl, creatinine ≤ 2.0 mg/dl) 8-No concomitant medications known to interfere with inflammatory and oxidative stress parameters such as NSAIDs, aspirin, and Cox-2 inhibitors	1-LVEF 2-Echocardiographic parameters 3-Serum levels of IL-6 4- Blood levels of ROS	"The protective effect of telmisartan is long-lasting, probably by ensuring a permanent (at least up to 18-month FU) defense against chronic or late-onset types of anthracycline-induced cardiotoxicity."
		placebo, 24	NA				
Jhorawat 2014 [32]	India	carvedilol, 27	12.5 mg	adriamycin	1-Patients diagnosed with lymphoreticular malignancy and planned for chemotherapy (CT) with a regimen containing ANT (ADR) between January 2008 and February 2009 2- Not to have undergone previous CT, or radiation therapy, and have no underlying coronary artery dr or previous DCM	1-LVEF 2-Result of Doppler examination	"Prophylactic use of carvedilol in patients receiving anthracycline protected systolic functions of the left ventricle. Carvedilol can be a potential drug that can ameliorate ADR- induced CMP."
		placebo, 27	NA				
Elitok 2014 [24]	Turkey	carvedilol, 40	12.5 mg	NA	1-Breast cancer patients who did not previously receive chemo or radiation therapy 2- Cardioprotective drug use, such as angiotensin-converting enzyme inhibitors, angiotensin receptor blockers, calcium channel blockers, statins, aldosterone receptor antagonists, and other beta-blockers 3- Who had no baseline cardiac dysfunction	1-LVEF	"These results indicate that carvedilol has a protective effect against the cardiotoxicity induced by ANT."
		placebo, 40	NA	NA			
Akpek 2014 [10]	Turkey	spironolactone, 43	25 mg	NA	1- Female patients with no prior breast cancer and/or prior anthracycline exposure history 2- LVEF > 50% 3- No prior use of ACE inhibitors, ARBs, or beta-blockers 4- Creatinine < 2 mg/Dl 5- No presence of chronic kidney failure 6- Potassium < 5.3 mg/Dl 7- No presence of adrenal gland diseases 8- No presence of severe liver failure 9- No presence of co-morbidities such as coronary heart disease, hypertension, AF, and valvular heart disease	1-LVEF 2- Haematological parameters	"spironolactone administration used simultaneously with anthracycline group chemotherapeutics protects both myocardial systolic and diastolic functions. Spironolactone can be used to protect against anthracycline-induced cardiotoxicity. "
		placebo, 40	NA	NA			
Chotenimitkhun 2015 [21]	USA	statin, 14	40–80 mg daily/10–20 mg daily	b-Blockers = 7 (50%), Angiotensin-converting enzyme inhibitors = 6 (43%), Angiotensin II receptor blockers = 4 (29%), Calcium channel blockers = 4 (29%), Diuretics 7 (50%)	1- Participants who were recruited from the hematology and oncology outpatient and inpatient facilities of the Comprehensive Cancer Center at Wake Forest Health Sciences 2- And were scheduled to receive Anth-bC	1-LVEF	" These data highlight the finding that individuals receiving statin therapy for prevention of cardiovascular disease may experience less deterioration in LVEF with early receipt of Anth-bC than individuals not receiving statins. Further studies with large numbers of participants are warranted to determine if statins protect against LVEF decline in patients receiving Anth-bC."
		non-statin, 37	NA	b-Blockers = 3(8%), Angiotensin-converting enzyme inhibitors = 4(11%), Angiotensin II receptor blockers = 0(0%), Calcium channel blockers = 1 (3%), Diuretics 1 (3%)			
Boekhout 2016 [18]	Germany	Candesartan,106	32 mg/d	NA	1-Aged 18 years or older; early-stage HER2-positive breast cancer 2-Completion of anthracycline-based adjuvant treatment 3- Performance status 2 or lower 4- LVEF at least 50% as measured by echocardiography or multiple gate acquisition radionuclide imaging 5- Creatinine clearance greater than 50 mL/min (by Cockcroft Gaultformula) 6- thyroid-stimulating hormone level between 0.5 and 3.9 MU/L or thyroid hormone-free thyroxine between 8 and 26 pmol/L 7- Systolic blood pressure between 100 and 180 mm Hg and diastolic blood pressure between 60 and 100 mm Hg 8- And first trastuzumab infusion received at least 3 weeks after day 1 of the last anthracycline infusion	1-LVEF	"The findings do not support the hypothesis that concomitant use of candesartan protects against a decrease in left ventricular ejection fraction during or shortly after trastuzumab treatment in early breast cancer. The ERBB2 germline Ala1170Pro single nucleotide polymorphism may be used to identify patients who are at increased risk of trastuzumab-related cardiotoxic effects"
		PLACEBO, 104	NA	NA			
Ahmad 2016	Iran	Carvedilol,30	6.25 mg	NA	1- Non menopausal women 2-No previous cardiac conditions (including ischemic heart disease, prolonged hypertension, and clinically important congenital or acquired valvular and myocardial diseases) or diabetes, no previous chemo/radiotherapy, taking no cardiac-related drugs, and not having other cancers	1-Echocardiographic parameters (LVEF & Strain rate)	"This study shows that carvedilol can prevent doxorubicin-induced cardiotoxicity. Whether this prophylaxis should be considered as the preferred method needs further investigation."
		Placebo,40	NA	NA			
Janbabai 2016 [31]	Iran	enalapril,34	5 mg twice daily	NA	1-Patients aged 21–74 years with (ECOG) performance status > 2 2-Normal sinus rhythm 3- And preserved LVEF at baseline echocardiography 4-With a newly diagnosed malignancy	1-LVEF	"prophylactic use of enalapril can be beneficial in preserving both systolic and diastolic function in cancer patients treated with ANTs."
		placebo,35	NA	NA			
Nabati 2017 [46]	Iran	Carvedilol,46	6.7 mg /day		1-Women with newly diagnosed breast cancer treated with ANT therapy	1-LVEF 2-Echocardiographic parameters	"Prophylactic use of carvedilol may inhibit the development of anthracycline-induced cardiotoxicity, even at low doses."
		Placebo,45	NA				
Pituskin 2017 [38]	Canada	Placebo, 30	NA	NA	1-Patients age > 18 years 2- With newly diagnosed HER2- overexpressing EBC (stage I to IIIA) 3- Planned adjuvant treatment with trastuzumab	1-Trastuzumab-mediated left ventricular remodeling	"Perindopril and bisoprolol protected against cancer therapy-related declines in LVEF; however, they did not prevent trastuzumab-mediated left ventricular remodeling."
		Perindopril, 33	8 mg/ daily	NA			
		Bisoprolol, 31	10 mg/daily	NA			
Avila 2018 [17]	Brazil	Carvedilol,96	3.125 mg twice-a-day	NA	1-Patients with HER2-negative breast cancer tumor status 2- Therapy that included anthracycline, cyclophosphamide	1-LVEF 2-BNP 3-TnI and diastolic dysfunction	“ In this largest clinical trial of β-blockers for the prevention of cardiotoxicity under contemporary ANT dosage, we noted a 13.5 –14.5% incidence of cardiotoxicity. In this scenario, carvedilol had no impact on the incidence of early onset of LVEF reduction. However, the use of carvedilol resulted in a significant reduction in troponin levels and diastolic dysfunction.”
		Placebo,96	NA	NA			
Wihandono 2021 [45]	Indonesia	lisinopril and bisoprolol, 37	10 mg/day	NA	1-Patients from 18–70 years old 2- Sinus rhythm and (LVEF ≥ 50%) 3- Diagnosed with locally advanced breast cancer and submitted to receive anthracycline-based neoadjuvant chemotherapy	1-LVEF	"Combined treatment with lisinopril and bisoprolol may prevent anthracycline-induced cardiotoxicity in patients with locally advanced breast cancer treated with anthracycline-based chemotherapy"
		Control,37	NA	NA			
Slowik 2020 [42]	Poland	Ramipril,48	10 mg/d	NA	1-Consecutive women with stages I–III BC 2- Who underwent breast surgery and were referred for adjuvant anthracycline therapy	1-LVEF 2-Troponin I 3-NT-proBNP 4-HF or cardiac death	"In relatively young women with BC without serious comorbidities, who received anthracyclines, 1-year treatment with ramipril exerts potentially protective effects on cardiotoxicity assessed with NT -proBNP levels."
		Control,48	NA	NA			
Sherafati 2019	Iran	carvedilol,27	6.25 mg twice daily	NA	1- Every HER2-positive breast cancer patient 2- Who was a candidate for receiving Herceptin therapy	1-LVEF 2-Echocardiographic parameters	"In conclusion, carvedilol may have beneficial effects in the prevention of cardiac dysfunction in patients receiving Herceptin but a larger study with a longer A follow-up period is recommended to determine this hypothesis more accurately"
		control, 38	NA	NA			
Nabati 2019 [37]	Iran	rosuvastatin,38	20 mg	NA	1- Women were 25 to 77 years old 2-Had been newly diagnosed with breast cancer 3- Had preserved LV systolic function in which the (LVEF) 55% 4-And had normal liver, renal, and hematological functions	1-LVEF 2-GLS 3-Cardiovascular mortality 4-hospitalization	"The present study showed that the prophylactic use of rosuvastatin may prevent the development of chemotherapy-induced cardiotoxicity"
		control,39	NA	NA			
Martha 2020 [44]	Indonesia	carvedilol, 40	2 × 6.25 mg daily	NA	1-Female patients with breast cancer older than 19 years old 2- Received FAC chemotherapy regimen and sinus heart rhythm	1-Left ventricular function	"Carvedilol did not prevent the decline of subclinical left ventricular function after the chemotherapy cycle. However, it may be more likely to benefit patients whose given a larger cumulative dose of anthracycline and have multiple risk factors."
		placebo, 40	NA	NA			
heck 2021 (from gulti 2016) [30]	Norway	Candesartan– metoprolol,28	32 mg/100 mg daily	Trastuzumab,7	1-Patients were adult women between 18 and 70 years of age 2- With LVEF ≥ 50% 3- Normal kidney function, and no serious comorbidities 4-Who after surgery for early breast cancer was scheduled for adjuvant anthracycline-containing therapy	1-LVEF 2-LV systolic dysfunction	"anthracycline-containing adjuvant therapy for early breast cancer was associated with a decline in LVEF during extended follow-up. Candesartan during adjuvant therapy did not prevent reduction in LVEF at 2 years but was associated with a modest reduction in left ventricular end-diastolic volume and preserved global longitudinal strain. These results suggest that a broadly administered cardioprotective approach may not be required in most patients with early breast cancer without preexisting cardiovascular disease"
		Candesartan– placebo,32	32 mg daily	Trastuzumab,7			
		Placebo–metoprolol,30	100 mg daily	Trastuzumab,6			
		placebo,40	NA	Trastuzumab,7			
Guglin 2019 [27]	USA	Carvedilol,156	NA	NA	1-Adult patients with normal LVEF and without major cardiovascular comorbidities	1-Serum biomarkers 2-Troponin I 3-BNP 4-LVEF	"In patients with HER2-positive breast cancer treated with trastuzumab, both lisinopril, and carvedilol prevented cardiotoxicity in patients receiving anthracyclines. For such patients, lisinopril or carvedilol should be considered to minimize interruptions of trastuzumab. (Lisinopril or Coreg CR in Reducing Side Effects in Women With Breast Cancer Receiving Trastuzumab"
		Lisinopril,158	NA	NA			
		Placebo,154	NA	NA			
Esfandbod 2021 [35]	Iran	Carvedilol,30	12.5 mg twice a day	NA	1-Patients recently diagnosed with HER2-positive breast cancer in stages I to IIIA 2- Candidates for trastuzumab therapy	1-Ejection Fraction (EF) 2- Pulmonary Artery Pressure (PAP)	"patients with HER2-positive breast cancer treated with trastuzumab, Carvedilol showed no significant protective effect on trastuzumab-induced cardiotoxicity."
		control, 30	NA				
GEORGAKOPOULOS 2010 [26]	Greece	Metoprolol,42	100 mg/twice/day)	NA	1-Patients with HL and NHL	1-Incidence of HF and subclinical cardiotoxicity 2- LVEF and Echocardiographic parameters	“ Study showed that metoprolol and enalapril do not reduce the risk of cardiotoxicity in patients treated with doxorubicin”
		control, 40	-				
		Enalapril,43	( 20 mg/ twice/day)				
GEORGAKOPOULOS 2019(10 years follow up) [25]	Greece	metoprolol,27	8.8 ± 3.1 mg	NA	1-Patients > 18 yr old 2- Eastern Cooperative Oncology Group Performance Status (ECOG PS) of 0 or 1 (Serum creatinine < 2.0 mg/dl 3-Normal sinus rhythm, (LVEF) > 50% Fraction shortening (FS) > 25% before CT	1- The occurrence of doxorubicin-induced clinical or subclinical long-term cardiotoxicity in lymphoma patients	"Clinical signs of heart failure were not seen in any patients and no statistically significant differences between baseline and 10-year findings were seen for echocardiographic variables. No evidence of long-term cardiotoxicity was seen and neither metoprolol nor enalapril offered an additional benefit."
		control, 26					
		enalapril,30	11 ± 0.68 mg				
Davis 2019 [22]	Canada	Eplerenone,22	50 mg daily	NA	1- Stage I-III breast cancer 2-Scheduled to undergo treatment with a doxorubicin-based chemotherapy regimen 3-Able to provide informed consent	1- change in Eʹavg at 6 months 2-LEVF	'concomitant administration of eplerenone for 6 months was not associated with significant differences in systolic or diastolic function compared with placebo in patients with early or locally advanced breast cancer treated with anthracycline-based chemotherapy''
		Placebo,22	NA	NA			
Rizka 2021 [40]	Indonesia	ACEI,15	10–5 mg daily	NA	1-Early and locally advanced breast cancer patients 2-Who received neoadjuvant chemotherapy anthracycline-based	1- Troponin	"The treatment group that received the ACEi intervention could prevent an increase of troponin levels after chemotherapy. Overall, it can be concluded that the consumption ACEi can inhibit the rise of Troponin"
		Control,15	NA	NA			
Farhani 2019	Iran	Carvedilol,36	6.25 mg twice a day	NA	1- HER2/neu-positive nonmetastatic patients with breast cancer 2- Who were treated with standard anthracyclines regimens 3- Who was candidated to receive trastuzumab	1-LVEF 2- GLS 3-and the strain rate of the LV systolic function [SRS])	" Concomitant carvedilol treatment with a maximum tolerable dose in patients with nonmetastatic HER2-positive breast cancer under treatment with trastuzumab might be effective in the reduction of systolic and diastolic echocardiographic findings other than the LVEF in patients with weak markers of heart failure. "
		Control,35	NA	NA			

*Abbreviations*: *NA* Not Applicable, *LVEF* Left Ventricular Ejection Fraction, *GLS* Global Length Strain, *BNP* B-type natriuretic peptide

### Baseline characteristics of the included population

The most common chemotherapy type was anthracycline. The most common cancer type in our included studies was breast cancer. Other types included lymphoma, leukemia, and others. The follow-up duration ranged from less than 1 month [43], and up to 120 months [24], with 6 months being the commonest follow-up duration. Table 2

**Table 2:** Baseline for included studies

N	Study ID	Study groups	Female, N (%)	Age (Mean ± Sd)	Cancer type	Type of chemotherapy	Accumulative dose of chemotherapy	Follow up duration	Definition of LV dysfunction	LVEF	NT-proBNP	Troponin
**1**	Gulati 2016 [28]	candestran + metoprolol	30 (100%)	50.0 ± 8.9	breast	trastuzumab	NA	NA	NA	62.2 ± 4.4	NA	NA
		candestran + placebo	32 (100%)	51.7 ± 10.7	breast	trastuzumab	NA			62.3 ± 5.3	NA	NA
		metoprolol + placebo	32 (100%)	50.5 ± 9.1	breast	trastuzumab	NA			63.5 ± 5.0	NA	NA
		placebo + placebo	32 (100%)	50.8 ± 9.2	breast	trastuzumab	NA			63.6 ± 4.1	NA	NA
**2**	Gupta 2017 [29]	enalapril	31 (70%)	8.85 ± 3.15	leukemia, lymphoma	anthracyclines	272.73 ± 78.62	6 months	decrease in LVEF of ≥ 20% from baseline to 6 months	62.25 ± 5.49	49.60 ± 35.97	0.01 ± 0.00
		placebo	30 (75%)	8.77 ± 2.86	leukemia, lymphoma	anthracyclines	263.64 ± 80.90			56.15 ± 4.79	98.60 ± 54.24	0.011 ± 0.003
**3**	Kalay 2006 [33]	carvedilol	22 (88%)	46.8 ± 14	breast, lymphoma, and other	anthracyclines	adriamycin = 525.3, epirubicin = 787.9	6 months	EF 50%	70.6 ± 8.0	NA	NA
		placebo	21 (84%)	49.0 ± 9.8	breast, lymphoma, other	anthracyclines	adriamycin = 513.6, epirubicin = 770.4			69.7 ± 7.3	NA	NA
**4**	Cardinale 2006 [20]	enalapril	33 (60%)	47 ± 11	breast, lymphoma, leukemia, Ewing's sarcoma	anthracyclines	332 ± 191	12 months	absolute decrease 10 percent units in rest LVEF associated with a decline below the normal limit value (50%).3,4	62.4 ± 3.5	NA	NA
		control	39 (67%)	44 ± 13	breast, lymphoma, leukemia, Ewing's sarcoma	anthracyclines	338 ± 167			62.8 ± 3.4	NA	NA
**5**	Georgakopoulos 2010 [26]	metoprolol	20 (48%)	51.0 ± 18.0	lymphoma	anthracyclines	387.5 ± 6.8	36 months	NA	67.7 ± 5.0	NA	NA
		enalapril	21 (49%)	47.4 ± 16.2	lymphoma	anthracyclines	373.1 ± 6.3			65.2 ± 7.1	NA	NA
		control	19 (47%)	49.1 ± 19.4	lymphoma	anthracyclines	386.4 ± 5.7			67.6 ± 7.1	NA	NA
**6**	Acar 2011 [15]	atorvastatin	12(60%)	53.7 ± 14.2	non-Hodgkin’s lymphoma, multiple myeloma, and leukemia	anthracyclines	NA	6 months	ejection fraction (EF) of < 50%	61.3 ± 7.9	NA	NA
		control	11(55%)	52.6 ± 17.6	non-Hodgkin’s lymphoma, multiple myeloma, and leukemia	anthracyclines	NA			62.9 ± 7.0	NA	NA
**7**	Salehi 2011 [41]	placebo	8 (36%)	43.50 ± 15.27	breast, lymphoma	anthracyclines	540.28 ± 31.17 mg/m2	4 months	NA	58.56 ± 3.62	NA	NA
		carvedilol 12.5 mg	7 (32%)	45.70 ± 14.16	breast, lymphoma	anthracyclines	531.50 ± 29.98 mg/m2			60.5 ± 5.07	NA	NA
		carvedilol 25 mg	5 (23%)	52.52 ± 11.00	breast, lymphoma	anthracyclines	521.14 ± 38.97 mg/m2			61.00 ± 7.06	NA	NA
**8**	Bosch 2013 [19]	enalapril and carvedilol	18 (40%)	49.7 ± 13.9	leukemia	anthracyclines	290 ± 189	6 months	LVEF declined below its normal limit of 50%	62 ± 5.9	19 ± 7.25	0.013 ± 0.008
		control	21(47%)	50.9 ± 13.2	leukemia	anthracyclines	241 ± 162			63 ± 5.9	21 ± 5.75	0.013 ± 0.010
**9**	Kaya 2013 [34]	Nebivolol	27(100%)	51.4 ± 9.4	breast	anthracyclines	epirubicin = 361 ± 88 adriamycin = 257 ± 29 cyclophosphamid = 2342 ± 870 5-Fluorouracil = 2325 ± 1015 docetaxel = 450 ± 0	6 months	LVEF < 45% at the end of the study	65.6 ± 4.8	65.42 ± 25.37	NA
		Placebo	18(100%)	50.5 ± 11.1	breast	anthracyclines	epirubicin = 348 ± 84 adriamycin = 235 ± 48 cyclophosphamid = 2269 ± 876 5-Fluorouracil = 2291 ± 1042 docetaxel = 337 ± 159			66.6 ± 5.5	64.09 ± 29.37	NA
**10**	Radulescu 2013 [39]	perindopril	36 (53%)	48.24 ± 12.2	lung, lymphoma, nasopharynx, breast, urinary bladder, and stomach	epirubicin	550 mg/m2	12 months	NA	58.48 ± 6.12	NA	NA
		control	38 (56%)	48.24 ± 12.2	lung, lymphoma, nasopharynx, breast, urinary bladder, and stomach	epirubicin	550 mg/m2			59.46 ± 7.12	NA	NA
**11**	Dessì 2013 [23]	telmisartan	19 (76%)	52.9 ± 9	breast, endometrial, NHL, NSCLC, ovarian, salivary gland	epirubicin	400 ± 30 mg/m2	18 months	LVEF under 55%	66 ± 5%	NA	NA
		placebo	18 (76%)	53 ± 10	breast, endometrial, NHL, NSCLC, ovarian, salivary gland	epirubicin	400 ± 30 mg/m2			66 ± 5%	NA	NA
**12**	Jhorawat 2014 [32]	Carvedilol	4 (14.8%)	43.89 ± 15.66	lymphoreticular malignancy	anthracycline	427.96 ± 124.36	6 months	a decline in LVEF of at least five percent to less than 55 percent with accompanying signs or symptoms of HF, or a decline in LVEF of at least 10 percent to below 55 percent without accompanying signs or symptoms	63.19 ± 7.22	NA	NA
		Placebo	9 (33.3%)	38.74 ± 18.36	lymphoreticular malignancy	anthracycline	395.07 ± 132.82			67.56 ± 5.98	NA	NA
**13**	Elitok 2014 [24]	carvedilol	40 (100%)	54.3 ± 9.3	breast cancer	anthracycline	535.6 mg/m2	6 months	NA	66 ± 6.1	NA	NA
		placebo	40 (100%)	52.9 ± 11.2	breast cancer	anthracyline	523.3 mg/m2			65 ± 4.5	NA	NA
**14**	Akpek 2014 [10]	Spironolactone	0(0%)	50.0 ± 10.8	breast cancer	anthracycline	adriamycin = 430.2 ± 52.2, epirubicin = 688.9 ± 136.0		NA	67.0 ± 6.1	81.33 ± 59.03	0.010 ± 0.014
		placebo	0(0%)	50.6 ± 10.1	breast cancer	anthracycline	adriamycin = 394.2 ± 71.9, epirubicin = 688.9 ± 120.5			67.7 ± 6.3	66 ± 12.03	0.011 ± 0.015
**15**	Chotenimitkhun 2015 [21]	statin	8(57%)	62 ± 2	breast cancer, leukemia, and lymphoma	anthracycline	Doxorubicin = 153 ± 35,Daunorubicin = 45 ± 25,Epirubicin = 36 ± 36	6 moths	NA	56.6% ± 1.4%	NA	NA
		non-statin	25(67%)	43 ± 2	breast cancer, leukemia, and lymphoma	anthracycline	Doxorubicin = 159 ± 20,Daunorubicin = 64 ± 22,Epirubicin = 9 ± 8			57.5% ± 1.4%	NA	NA
**16**	Boekhout 2016 [18]	Candesartan	103(100%)	50 ± 2.33	breast cancer	trastuzumab	440 mg/m2	21 months	a decline in LVEF of > 15% or a decrease below the absolute value of 45%	60 ± 1.5	27.59 ± 5.26	0.014 ± 2.17
		placebo	103(100%)	51 ± 2.17	breast cancer	trastuzumab	440 mg/m2			61 ± 1.5	31.6 ± 5.86	13 ± 1.83
**17**	Ahmad 2016	Carvedilol	30 (100%)	42 ± 6.3	breast cancer	Doxorubicin	240 mg/m 2	NA	a A 10–50% drop was considered an indication of discontinuation of the chemotherapy	59.41 ± 4.2	NA	NA
		placebo	40(100%)	39.9 ± 6.3	breast cancer	Doxorubicin	240 mg/m 2			61.31 ± 3.21	NA	NA
**18**	Janbabai 2016 [31]	placebo	31 (88.6%)	47.06 ± 12.39	Breast, Wilms tumor, Lung cancer, Bone sarcoma, Hodgkin’s lymphoma	Anthracycline	366.69 ± 21.76	6 months	LVEF < 45%	NA	NA	0.065 ± 0.0175
		enalapril	33 (97.1%)	47.76 ± 11.81	Breast, Wilms tumor, Lung cancer, Bone sarcoma, Hodgkin’s lymphoma	Anthracycline	363.34 ± 34.87			59.61 ± 5.70	NA	0.016 ± 0.011
**19**	Nabati 2017 [46]	Placebo	45(100%)	47.10 ± 12.17	breast cancer	Anthracyclines	doxorubicin = 359.91 ± 27.13 cyclophosphamide = 3390.59 ± 234.67	6 months	NA	61.13 ± 4.97	NA	0.146 ± 0.055
		carvedilol	46(100%)	47.57 ± 8.75	breast cancer	Anthracyclines	doxorubicin = 348.56 ± 40.34 0.141 cyclophosphamide = 3653.51 ± 405.64			58.72 ± 4.69	NA	0.073 ± 0.022
**20**	Pituskin 2017 [38]	placebo	30(100%)	51 ± 7	breast cancer	trastuzumab		24-month	change in indexed left ventricular end-diastolic volume and LVEF	61 ± 5	NA	NA
		perindopril	33(100%)	50 ± 8	breast cancer	trastuzumab				62 ± 5	NA	NA
		Bisoprolol	31(100%)	53 ± 10	breast cancer	trastuzumab				62 ± 4	NA	na
**21**	Avila 2018 [17]	carvedilol	96(100%)	50.8 ± 10.10	breast cancer	Anthracycline	240 mg/m2	6 months	≥ 10% reduction in LVEF	64.8 ± 4.7	16 ± 2.79	0.005 ± 0
		placebo	96(100%)	52.9 ± 9.05	breast cancer	Anthracycline	240 mg/m2			65.2 ± 3.6	12 ± 2.5	0.005 ± 0
**22**	Wihandono 2021 [45]	lisinopril and bisoprolol	26(100%)	44,5 ± 7,7	breast cancer	Anthracyclines	579.48 ± 65.10	5 months	change from baseline LVEF	65.77 ± 4.56	NA	NA
		placebo	25(100%)	50,8 ± 7,39	breast cancer	Anthracyclines	557.50 ± 47.76			65.44 ± 4.55	NA	NA
**23**	Slowik 2020 [42]	ramipril	48(100%)	45.67 ± 12.23	breast cancer	Anthracycline	(doxorubicin dose ≥ 250 mg/m2)	12 months	decrease in left ventricular LVEF, elevated biomarker levels, and/or HF or cardiac death	67.33 ± 3.82	NA	NA
		placebo	48(100%)	45.67 ± 8.41	breast cancer	anthracycline				66 ± 3.82	NA	NA
**24**	Sherafati 2019	Carvedilol	27(100%)	46.5	breast cancer	trastuzumab	NA	3 months	(LVEF < 50% or a decrease in EF over 10%)	56.3 ± 3.7	NA	NA
		control	38(100%)		breast cancer	trastuzumab	NA			56.9 ± 3.6	NA	NA
**25**	Nabati 2019 [37]	placebo	39(100%)	50.74 ± 12.440	breast cancer	anthracycline	337.83 ± 42.60	6 months	changes in the LVEF and the global longitudinal strain (GLS)	55.10 ± 5.09	NA	NA
		Rosuvastatin	38(100%)	47.74 ± 9.70	breast cancer	anthracycline	339.44 ± 39.00			55.05 ± 4.84	NA	NA
**26**	Martha 2020 [44]	carvedilol	40(100%)	48 ± 10	breast cancer	5-fluorouracil, Adriamycin, Cyclophosphamide (FAC)	consisted of 5-fluorouracil 600 mg/m2, Adriamycin 60 mg/m2, and cyclophosphamide 600 mg/m2	< 1 month	reduction of LVEF > 10%, to a value of < 53%	NA	NA	NA
		control	40(100%)	48 ± 8	breast cancer	5-fluorouracil, Adriamycin, Cyclophosphamide (FAC)				NA	NA	NA
**27**	Heck 2021 (from gulti 2016) [30]	Candesartan–metoprolol	28(100%)	50 ± 9	breast cancer	anthracyclines	NA	24 months	defined as LVEF < 53% in combination with either an absolute decrease of > 10% in LVEF as determined by CMR or clinical heart	NA	NA	NA
		Candesartan–placebo	32(100%)	52 ± 11	breast cancer		NA			NA	NA	NA
		Placebo–metoprolol	30(100%)	51 ± 9	breast cancer		NA			NA	NA	NA
		Placebo–placebo	30(100%)	51 ± 9	breast cancer		NA			NA	NA	NA
**28**	Guglin 2019 [27]	carvedilol	156(100%)	51.58 ± 10.93	breast cancer	anthracyclines	NA	24 months	decrease in LVEF of > 10% in patients whose LVEF is > 50%; or a drop in LVEF of at least 5% from baseline in patients whose LVEF decreases to < 50%	62.55 ± 6.61	38.3 ± 40.2	NA
		Lisinopril	158(100%)	50.58 ± 10.91	breast cancer	anthracyclines	NA			62.97 ± 6.18	31.3 ± 16.7	NA
		Placebo	154(100%)	51.11 ± 10.32	breast cancer	anthracyclines	NA			62.24 ± 6.09	37.5 ± 39.4	NA
**29**	Esfandbod 2021 [35]	Carvedilol	30(100%)	47.6 ± 9.64	breast cancer	Trastuzumab	NA	12 months	1- Global LVEF decrease 2- Interventricular LVEF decrease 3- 5% or more decrease from basal LVEF or EF < 55% in symptomatic patients 4- 10% or more decrease from basal LVEF or EF < 50% in asymptomatic patients	55 ± 1.03	NA	NA
		Placebo	30(100%)	46.2 ± 8.59	breast cancer	Trastuzumab	NA			54.9 ± 0.45	NA	NA
**30**	GEORGAKOPOULOS 2019(10 years follow up) [25]	Metoprolol	20 (47%)	51.0 ± 18.0	Lymphoma	Anthracyclines	NA	120 months	difference of 10% between the LVEF values at each visit and the baseline value, and LVEF < 50%	65.7 ± 5.0	NA	NA
		Enalapril	21 (49%)	47.4 ± 16.2	Lymphoma	Anthracyclines	NA			65.2 ± 7.1	NA	NA
		control	19(47%)	49.1 ± 19.4	Lymphoma	Anthracyclines,	NA			67.6 ± 7.1	NA	NA
**31**	Farahani 2019 [36]	Carvedilol	36(100%)	57.3 ± 7.3	breast cancer	Trastuzumab		3 months	LVEF reductions of greater than 10% to less than 50% or LVEFs of less than 45%	54.93 ± 4.26	NA	NA
		placebo	36(100%)	57.4 ± 8.8	breast cancer	Trastuzumab				54.32 ± 5.32	NA	NA
**32**	Davis 2019 [22]	Eplerenone	22(100%)	53.9 ± 2.0	breast cancer	Anthracycline	240 mg/m2	6-month	a decline in LVEF of 10% to an absolute value of < 50%;	63.2 ± 3.9	28.5 ± 14, 36	NA
		placebo	22(100%)	49.1 ± 12.8	breast cancer	Anthracycline				64.4 ± 4.2	31.0 ± 9, 53	NA
**33**	Rizka 2021 [40]	ACE Is	15(100%)	55 ± 7.387	breast cancer	anthracycline	NA	NA	NA	NA	NA	0.0120 ± 0.0077
		control	15(100%)	48.93 ± 9.573	breast cancer	anthracycline	NA			NA	NA	0.0127 ± 0.0059

*Abbreviations*: *ACEI* Angiotensin-converting enzyme inhibitor, *LVEF* Left ventricular Ejection Fraction, *NA* Not Applicable, *BNP* B-type natriuretic peptide

### Risk of bias

**Fig. 2:**
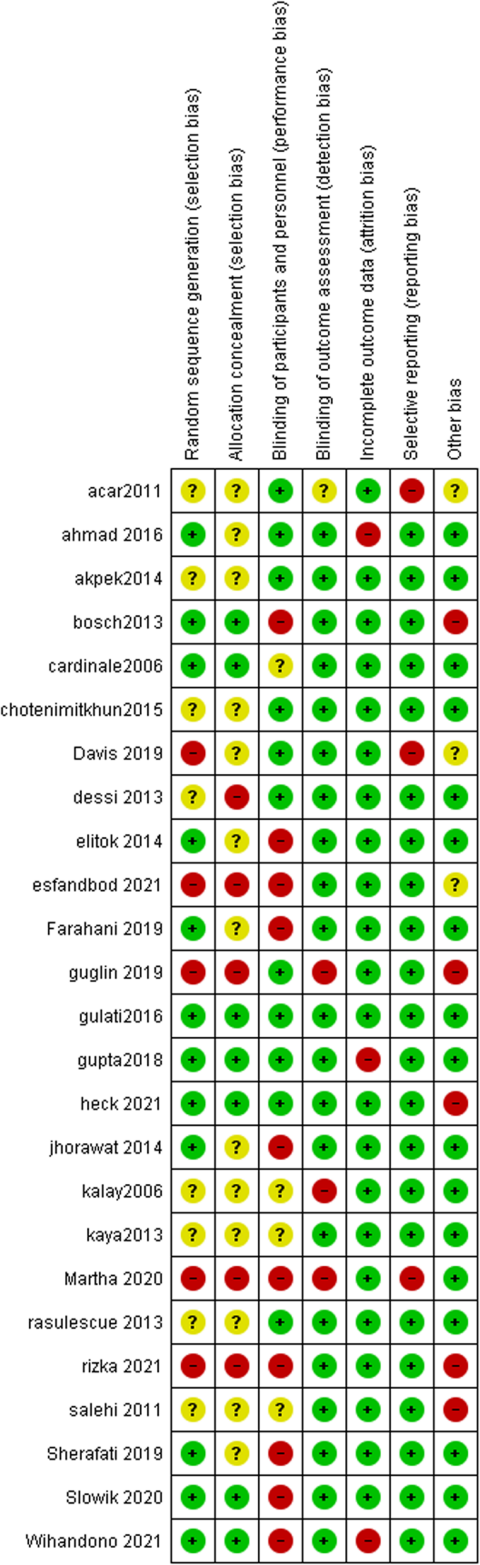
Risk of bias graph

The overall quality of the included trials ranged from moderate to high. We detected a high risk of selection bias in five RCTs, performance bias in ten RCTs, and reporting and attrition biases in two RCTs. There was unclear selection bias in eight studies, unclear performance bias in four studies, and unclear detection bias in one study. The remaining RCTs have a low risk of bias. Figure 2.

**Fig. 3:**
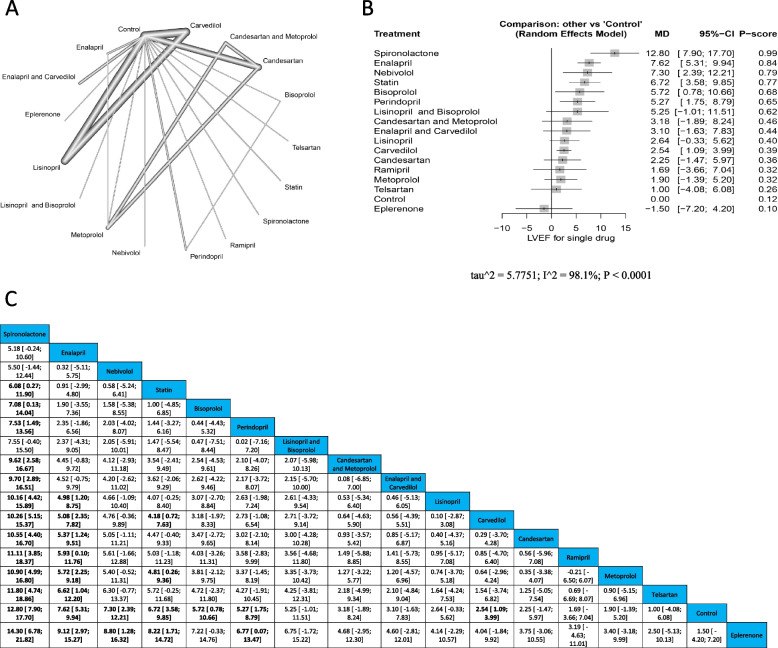
LVEF **A** Network graph showing direct evidence between the assessed drugs. **B** A forest plot comparing all drugs with control; P-score used for ranking. **C** The league table represents the network meta-analysis estimates for all drugs’ comparisons

**Fig. 4:**
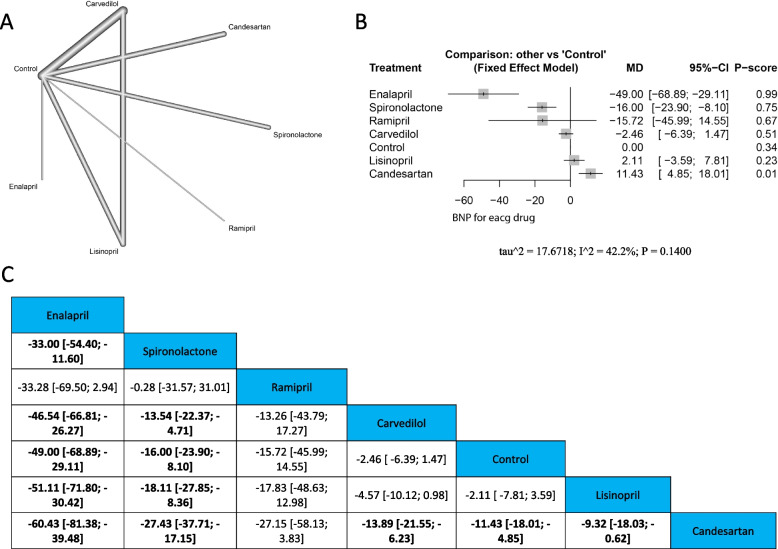
BNP **A** Network graph showing direct evidence between the evaluated drugs. **B** A forest plot comparing all drugs with control. **C** The league table represents the network meta-analysis estimates for all drugs' comparisons

**Fig. 5:**
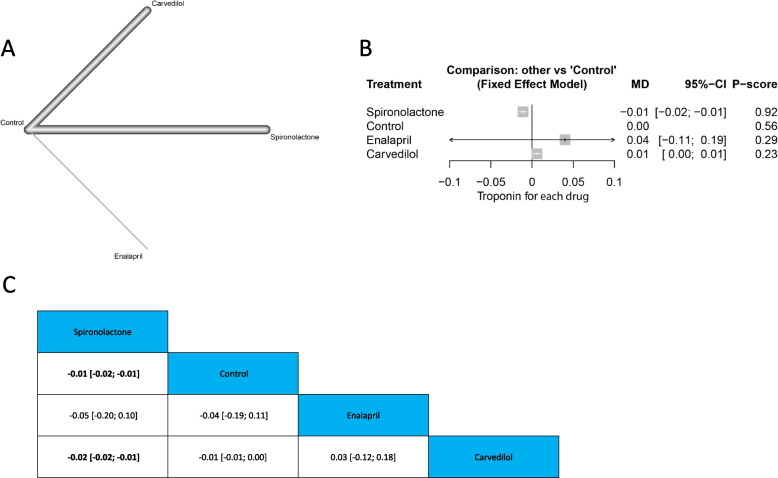
Troponin **A** Network graph showing direct evidence between the evaluated drugs. **B** A forest plot comparing all drugs with control. **C** The league table represents the network meta-analysis estimates for all drugs' comparisons

**Fig. 6:**
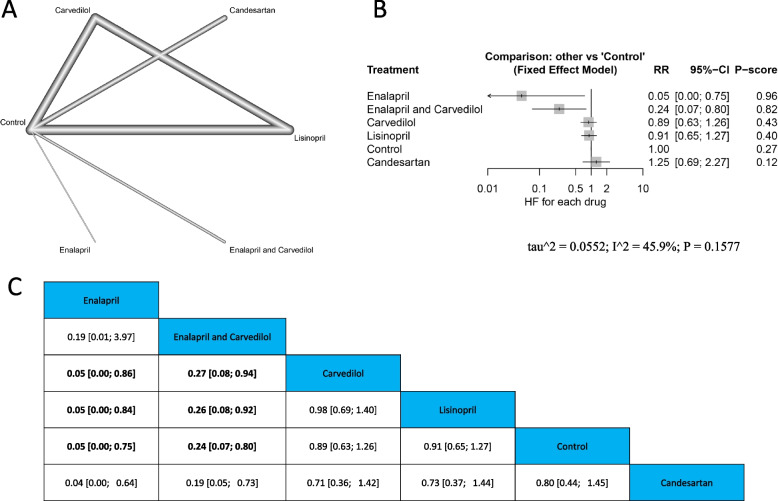
Heart failure **A** Network graph showing direct evidence between the evaluated drugs. **B** A forest plot comparing all drugs with control. **C** The league table represents the network meta-analysis estimates for all drugs’ comparisons

### Outcomes

#### LVEF

##### Single drugs

The pooled estimate showed the highest significant improvement with spironolactone (MD = 12.80, 95% CI [7.90; 17.70]), followed by Enalapril (MD = 7.62, 95% CI [5.31; 9.94]), Nebivolol (MD = 7.30, 95% CI [2.39; 12.21]), Statin (MD = 6.72, 95% CI [3.58; 9.85]), Bisoprolol (MD = 5.72, 95% CI [0.78; 10.66]), Perindopril (MD = 5.27, 95% CI [1.75; 8.79]), and Carvedilol (MD = 2.54, 95% CI [1.09; 3.99]) compared to control. Other drugs revealed no significant improvements compared with the control. These results revealed heterogeneity (*p* < 0.0001, I^2^ = 98.1%). Both spironolactone and enalapril showed significantly better protection from dropping LVEF than Lisinopril (MD = 10.16, 95% CI [4.42; 15.89], & MD = 4.98, 95% CI [1.20; 8.75] respectively), Carvedilol (MD = 10.26, 95% CI [5.15; 15.37], & MD = 5.08, 95% CI [2.35; 7.82] respectively), Candesartan (MD = 10.55, 95% CI [4.40; 16.70], & MD = 5.37, 95% CI [1.24; 9.51] respectively), Ramipril (MD = 11.11, 95% CI [3.85; 18.37], & MD = 5.93, 95% CI [0.10; 11.76] respectively), Metoprolol (MD = 10.90, 95% CI [4.99; 16.80], & MD = 5.72, 95% CI [2.25; 9.18] respectively), Telmisartan (MD = 11.80, 95% CI [4.74; 18.86], & MD = 6.62, 95% CI [1.04; 12.20] respectively), and Eplerenone (MD = 14.30, 95% CI [6.78; 21.82], & MD = 9.12, 95% CI [2.97; 15.27] respectively). Also, spironolactone revealed significant improvements in decreasing reduction of EF compared to Statin (MD = 6.08, 95% CI [0.27; 11.90]), Bisoprolol (MD = 7.08, 95% CI [0.13; 14.04]), Perindopril (MD = 7.53, 95% CI [1.49; 13.56]), Candesartan and Metoprolol (MD = 9.62, 95% CI [2.58; 16.67]), Enalapril and Carvedilol (MD = 9.70, 95% CI [2.89; 16.51]). Statin was significantly better than Carvedilol (MD = 4.18, 95% CI [0.72; 7.63]), and Metoprolol (MD = 4.81, 95% CI [0.26; 9.36]). Finally, Nebivolol, Statin, and Perindopril were better than Eplerenone (MD = 8.80, 95% CI [1.28; 16.32], MD = 8.22, 95% CI [1.71; 14.72], and MD = 6.77, 95% CI [0.07; 13.47] respectively). Figure (3) (A) Network graph showing direct evidence between the assessed drugs. (B) A forest plot comparing all drugs with control; P-score used for ranking. (C) The league table represents the network meta-analysis estimates for all drugs' comparisons.

There was no publication bias as seen from the funnel plot using the Egger test (*P* = 0.4628). Supplementary Fig. 1.

##### Drug families

The pooled estimate showed the highest significant improvement EF with a statin (MD = 6.72, 95% CI [3.36; 10.08]), followed by an aldosterone antagonist (MD = 6.58, 95% CI [2.58; 10.58]), then ACEI (MD = 5.36, 95% CI [3.71; 7.01]), and beta-blocker (MD = 3.05, 95% CI [1.73; 4.38]), when compared with control. These results showed heterogeneity (*p* < 0.001, I^2^ = 98.3%). There were no significant differences between ACEI & beta blocker, ARBS & beta blocker, and ARBS with the control. Both statin and ACEI were better compared with beta-blocker (MD = 3.67, [0.06; 7.28], and (MD = 2.30, 95% CI [0.43; 4.18] respectively. Supplementary Fig. (2) (A) Network graph revealing direct evidence between the assessed drug families. (B) A forest plot comparing all drug families with control. (C) The league table represents the network meta-analysis estimates for all drug families' comparisons.

There was no publication bias as seen from the funnel plot using the Egger test (*P* = 0.1990). Supplementary Fig. 3.

#### BNP

##### Single drugs

The analysis demonstrated the highest significant decrease with Enalapril (MD =  − 49.00, 95% CI [− 68.89; − 29.11]), followed by spironolactone (MD =  − 16.00, 95% CI [− 23.9; − 8.10]), compared with control. On the other hand, candesartan had a significant increase compared with the control (MD = 11.43, 95% CI [4.85; 18.01]). Other drugs revealed no significant differences compared with the control. The results were homogenous (*p* = 0.140, I^2^ = 42.2%). Enalapril was better than spironolactone (MD =  − 33.00, 95% CI [− 54.40; − 11.60]). However, both enalapril and spironolactone had significant reductions compared with Carvedilol (MD = -46.54, 95% CI [-66.81; -26.27], & (MD = -13.54, 95% CI [-22.37; -4.71]), Lisinopril (MD = -51.11, 95% CI [-71.80; -30.42] respectively), & (MD = -18.11, 95% CI [-27.85; -8.36] respectively), and candesartan (MD = -60.43, 95% CI [-81.38; -39.48], & (MD = -27.43, 95% CI [-37.71; -17.15] respectively). Moreover, carvedilol, control, and lisinopril showed significant reductions than candesartan (MD = -13.89, 95% CI [-21.55; -6.23], MD = -11.43, 95% CI [-18.01; -4.85], MD = -9.32, 95% CI [-18.03; -0.62] respectively). Figure (4) (A) Network graph showing direct evidence between the evaluated drugs. (B) A forest plot comparing all drugs with control. (C) The league table represents the network meta-analysis estimates for all drugs' comparisons.

##### Drug families

The pooled estimate showed the highest significant decrease in BNP with ACEI (MD =  − 37.67, 95% CI [− 56.81; − 18.53]), followed by aldosterone antagonist (MD =  − 16.00, 95% CI [− 30.92; − 1.08]) when compared with control. However, beta-blocker and ARBS demonstrated no significant variations compared with the control. These results were heterogeneous (*p* = 0.0368, I^2^ = 60.9%). Both ACEI and aldosterone antagonists had more significant reductions than ARBS (MD = -49.10 [-72.97; -25.23], & MD = -27.43 [-48.06; -6.80] respectively. Moreover, ACEI was better than beta-blocker (MD = -35.90, 95% CI [-56.80; -15.00]). Supplementary Fig. (4) (A) Network graph revealing direct evidence between the assessed drug families. (B) A forest plot comparing all drug families with control. (C) The league table represents the network meta-analysis estimates for all drug families' comparisons.

#### Troponin

##### Single drugs

The analysis showed a significant reduction with spironolactone compared with control (MD =  − 0.01, 95% CI [− 0.02; − 0.01]), and compared with carvedilol (MD = -0.02, 95% CI [-0.02; -0.01]). Figure (5) A, B and C.

##### Drug families

The analysis showed a significant reduction in troponin with aldosterone antagonist compared with control (MD =  − 0.01, 95% CI [− 0.02; − 0.01]), and with beta-blocker (MD =  − 0.02, 95% CI [− 0.02; − 0.01]). Supplementary Fig. (5) A, B and C.

#### Heart failure

##### Single drugs

Enalapril had the lowest significant risk of incident heart failure (RR = 0.05, 95% CI [0.00; 0.75]), followed by Enalapril and Carvedilol (RR = 0.24, 95% CI [0.07; 0.8]), compared with control. The results were homogenous (*p* = 0.1577, I^2^ = 45.9%). Also, Enalapril, and Enalapril & Carvedilol showed lower HF risk compared to Carvedilol ((RR = 0.05, 95% CI [0.00; 0.86], & (RR = 0.27, 95% CI [0.08; 0.94] respectively), and Lisinopril (RR = 0.05, 95% CI [0.00; 0.84], & RR = 0.26, 95% CI [0.08; 0.92] respectively). Figure (6) A, B and C.

##### Drug families

The pooled estimate indicated non-significant differences between different drug families and control, and with each other. The results showed heterogeneity (*p* = 0.0453, I^2^ = 62.7%). Supplementary Fig. (6) A, B and C.

## Discussion

This updated network meta-analysis examined the findings of 33 RCTs including 3,285 patients to investigate the effects of cardiac drugs used for the prevention or treatment of chemotherapy-induced cardiotoxicity and cardiac dysfunction.

In a single drug comparison, spironolactone demonstrated the greatest improvement of LVEF compared to a control, followed by enalapril, nebivolol, statin, bisoprolol, perindopril, and carvedilol. Spironolactone showed a significant troponin reduction when compared to the control. Enalapril demonstrated the greatest significant decrease in BNP compared to the control, followed by spironolactone.

According to the drug class, statins demonstrated the greatest improvement in decreasing reduction in LVEF compared to control, followed by aldosterone antagonists, ACEi, and beta-blockers. Aldosterone antagonists showed the highest reduction in troponin compared to the other drug classes. ACEI demonstrated the greatest reduction in BNP compared to a control, followed by Aldosterone antagonists. With regards to the risk of clinical heart failure, enalapril showed a lower risk compared to carvedilol, lisinopril, and control. There was no significant difference between the drug classes in this regard.

A previous systematic review and network meta-analysis of RCTs examined the role of commonly used cardioprotective in chemotherapy-induced cardiotoxicity. They concluded that the drug classes aldosterone antagonists, ACEi, statins, and beta-blockers improved LV systolic function. In the analysis of single drugs or drug combinations, they noted a significant cardioprotective effect with spironolactone, enalapril, and statin and no effect with ARB [47].

In agreement with the findings of this network meta-analysis, we found that statins, aldosterone antagonists, ACEi, and beta-blocker improved LVEF while ARBS had no significant effect. We also noted comparable results with significant improvements in LV systolic function with spironolactone, enalapril, and statin showed when compared to a control.

However, in contrast to Liu et al., our single drug analysis found a significant difference between spironolactone, enalapril, and statins. We also noted a significant LVEF improvement with nebivolol and bisoprolol, contrary to the findings of the previous analyses [47]. Our findings on statins agree with a previous meta-analysis that associated statins with a reduced cardiotoxicity risk following anthracycline and/or trastuzumab exposure [48].

According to the results of our analysis, spironolactone is an effective drug for cardiotoxicity prevention, with significant effects on LVEF function, consistent with previous research [11, 49]. Prevention and treatment of anthracycline-induced cardiotoxicity with enalapril, carvedilol, and statin are approaches to decreasing cardiovascular risk [48].

The efficacy of spironolactone may be explained by the beneficial effects of aldosterone antagonists on cardiac remodeling following myocardial damage due to antifibrotic and antioxidant effects [49]. Similarly, statins have anti-inflammatory and anti-oxidative effects and reduce cardiac remodeling. They may also exert an effect on anthracycline and/or trastuzumab-induced damage pathways of cardiotoxicity, through the inhibition of small Ras homologous (Rho) GTPases which reduce topoisomerase II inhibition and the subsequent generation of ROS [48].

Previously, cardiotoxicity has been heterogeneously defined [50–52] with alterations in its concept over time [53]. The most widely used definition is related to LVEF changes [53]. Therefore, our primary outcome was LVEF. We note that a recent meta-analysis [54] concluded that the LVEF impairment's magnitude caused by modern anthracycline treatments, the most studied agent across the RCTs included in our meta-analysis, was less than previously reported. This suggests that LVEF may not be the optimal surrogate measure of cardiotoxicity [54], especially as by the time cardiotoxicity is detected by this method, significant LV dysfunction has already occurred. Furthermore, significant reductions in LVEF are not always reflected clinically by NYHA III or IV symptoms and the clinical significance and impacts of these subclinical changes on management are yet to be fully understood [55].

In a study of 500 HER2 + patients treated with trastuzumab, 27% of patients were diagnosed with cardiotoxicity (symptomatic HF or asymptomatic drop in LVEF) however this only led to treatment discontinuation in 5% of patients. Among those with asymptomatic heart failure, as determined by LVEF, comparable rates of complete recovery of cardiac function were noted between those in whom trastuzumab was discontinued and those whose treatment was discontinued, and those whose treatment was not interrupted. In the same study, two patients developed symptomatic heart failure with radiographic signs despite preserved LVEF [56]. This data suggests that LVEF may not be an optimal surrogate measure of cardiotoxicity and further research is necessary to determine if an alternate threshold is appropriate for whether chemotherapy should be interrupted or completed in the presence of cardiotoxicity.

Cardiac biomarkers such as NT- proBNP, Cardiac Troponin I and Troponin T, and CK-MB have been used to assess chemotherapy-induced cardiotoxicity [57, 58], and may provide a more timely and accurate measure of cardiac dysfunction than LVEF. The literature points towards a possible association between these biomarkers and chemotherapy-induced cardiotoxicity. NT-proBNP has shown promise as an early marker of subclinical late toxicity in pediatric patients treated for cancer [59, 60].

Cardiac biomarkers may also have utility in the risk stratification of patients’ pre-treatment. In one study of 450 female patients receiving treatment with trastuzumab, an elevated baseline troponin was significantly associated with a fourfold risk of developing trastuzumab-related cardiac dysfunction (TRCD) [61]. Serial troponin measurement may also have a utility in risk stratification. In a study of 700 patients, those who had an early troponin I rise (within 72 h of treatment) that persisted one month later, experienced a greater degree of cardiac impairment and a higher incidence of cardiac events than those with no or a transient increase [62]. A follow-up study of 470 patients, demonstrated the possible utility of biomarkers for early risk stratification, in patients that have no or equivocal echocardiographic changes. Those with an early troponin rise (> 0.07 mg/L) were randomized to receive enalapril or standard of care. No reduction in LVEF was noted in those who received ACEi, compared to 43% in the standard of care. The outcomes of our meta-analysis included BNP and troponin levels, with reductions in these markers agreeing with drugs that had the greatest effect on LVEF. The prognostic value and clinical significance of biomarker reduction in response to medical treatment for CTRCD is an area that requires further research.

The strengths of the current analysis are the inclusion of a high number of RCTs, covering 3285 patients across diverse geographical areas, the largest sample size for a study of CTRCD; and contemporaneous data with 12 RCTs from between 2019 and 2021. Limitations include the heterogeneity of the studies analyzed, with different cancer types, chemotherapy regimens, and varying durations of follow-up.

As future priorities for research in this area, we recommend large-scale RCTs, with a long duration of follow-up, that aim to offer direct comparisons between the most common cardioprotective drugs within a defined study population of patients e.g. patients with solid tumors. Further, we recommend that future research focus on the elucidation of more sensitive surrogates or direct measures of cardiotoxicity, such as myocardial strain imaging or biomarker assays [55].

## Conclusion

According to our single drug analysis, spironolactone demonstrated the most significant improvement of LVEF, the highest troponin reduction, and the 2^nd^ most significant BNP decrease. Enalapril demonstrated the greatest BNP reduction and the 2^nd^ greatest improvement in LVEF. According to drug class, statins demonstrated the greatest improvement in LVEF. Nebivolol, bisoprolol, perindopril, and carvedilol also showed positive results in terms of LVEF significant improvements. In agreement with previous meta-analyses, ARBs appear to have no clear role to play in chemotherapy-induced cardiac failure. Further studies that focus on specific chemotherapy classes that have longer follow-up duration, time-to-event analysis, and mortality analysis will be needed to help elucidate the full potential of cardioprotective agents and help determine which to use for each demographic.

## Supplementary Information

**Additional file 1: Supplementary Figure 1.** Funnel plot of publication bias for LVEF (single drug).

**Additional file 2: Supplementary Figure 2.** LVEF (A) Network graph revealing direct evidence between the assessed drug families. (B) A forest plot comparing all drug families with control. (C) The league table represents the network meta-analysis estimates for all drug families' comparisons.

**Additional file 3: Supplementary Figure 3.** Funnel plot of publication bias for LVEF (drug families).

**Additional file 4: Supplementary Figure 4.** BNP (A) Network graph revealing direct evidence between the assessed drug families. (B) A forest plot comparing all drug families with control. (C) The league table represents the network meta-analysis estimates for all drug families' comparisons.

**Additional file 5: Supplementary Figure 5.** Troponin (A) Network graph revealing direct evidence between the assessed drug families. (B) A forest plot comparing all drug families with control. (C) The league table represents the network meta-analysis estimates for all drug families' comparisons.

**Additional file 6: Supplementary Figure 6.** Heart Failure (A) Network graph revealing direct evidence between the assessed drug families. (B) A forest plot comparing all drug families with control. (C) The league table represents the network meta-analysis estimates for all drug families' comparisons.

**Additional file 7: Supplementary Table 1.** Search strategy.

## Data Availability

Not applicable.
