# Supplementary figures and images for: Efficacy and safety of cardioprotective drugs in chemotherapy-induced cardiotoxicity: an updated systematic review & network meta-analysis
Source: Cardiooncology. 2023 Feb 18;9:10. doi: 10.1186/s40959-023-00159-0 (PMC9938608; doi:10.1186/s40959-023-00159-0)

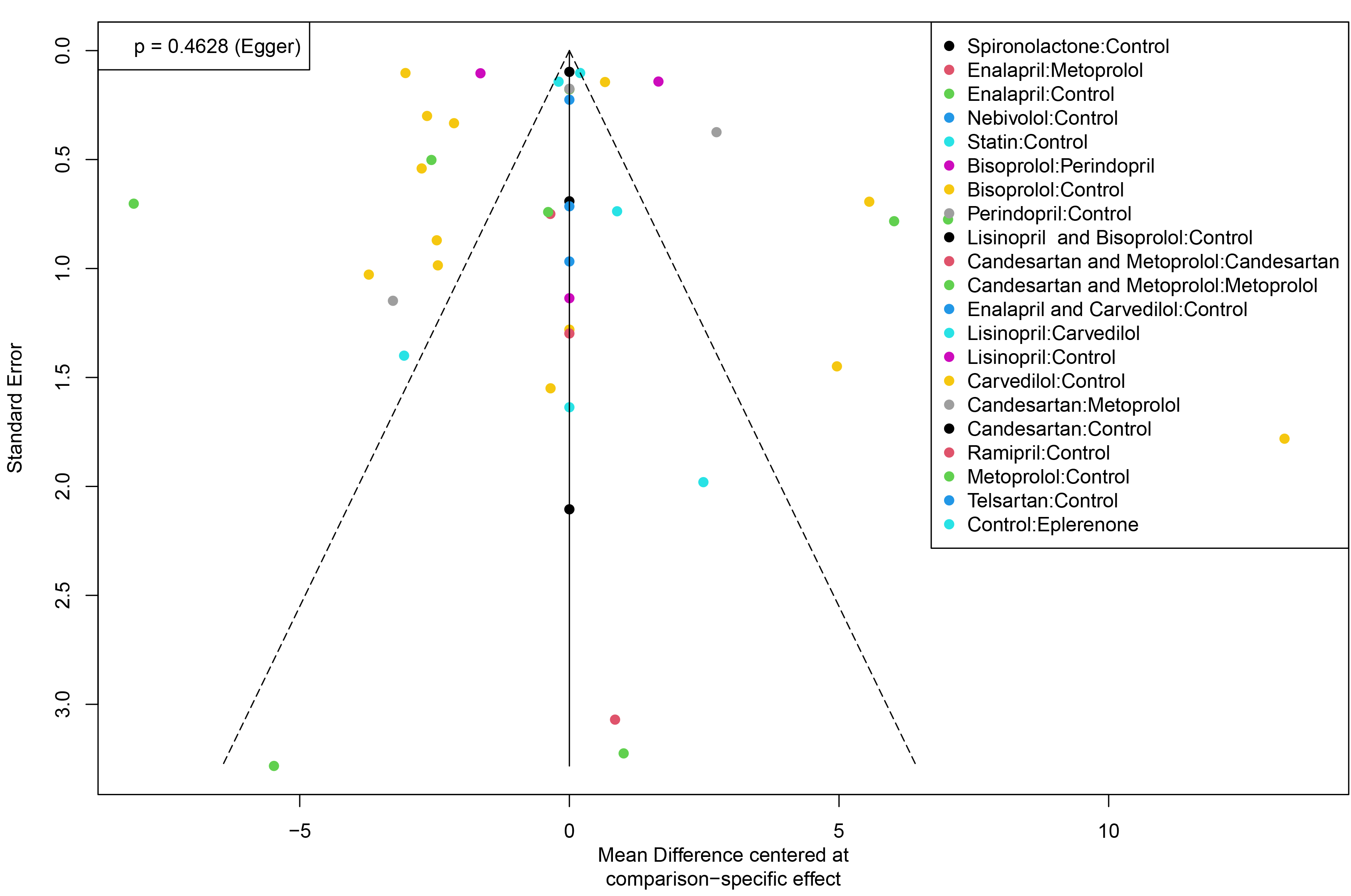

Supplement: Supplementary file 1 — Additional file 1: Supplementary Figure 1. Funnel plot of publication bias for LVEF (single drug). [file 40959_2023_159_MOESM1_ESM.tif]

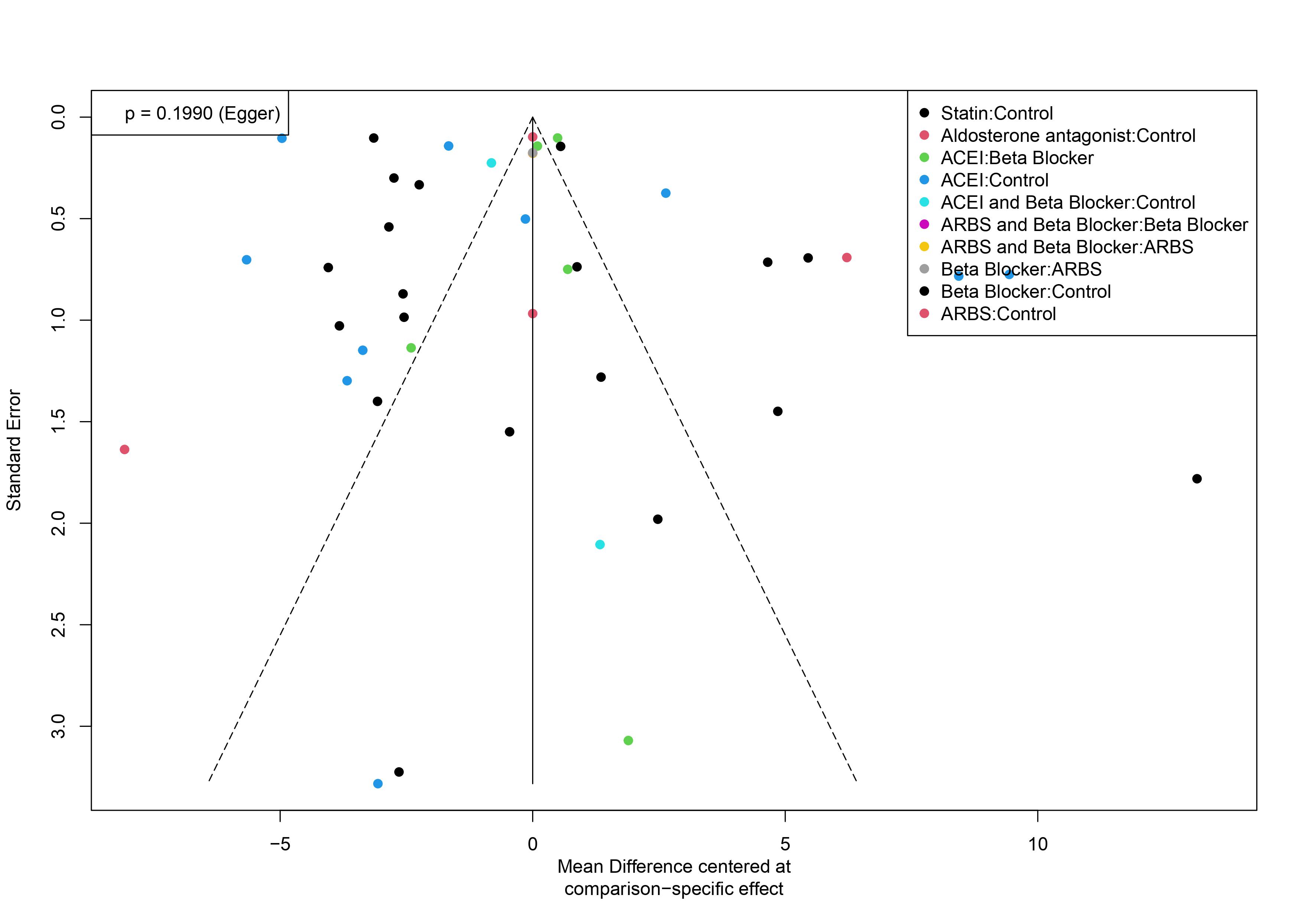

Supplement: Supplementary file 3 — Additional file 3: Supplementary Figure 3. Funnel plot of publication bias for LVEF (drug families). [file 40959_2023_159_MOESM3_ESM.jpg]

A

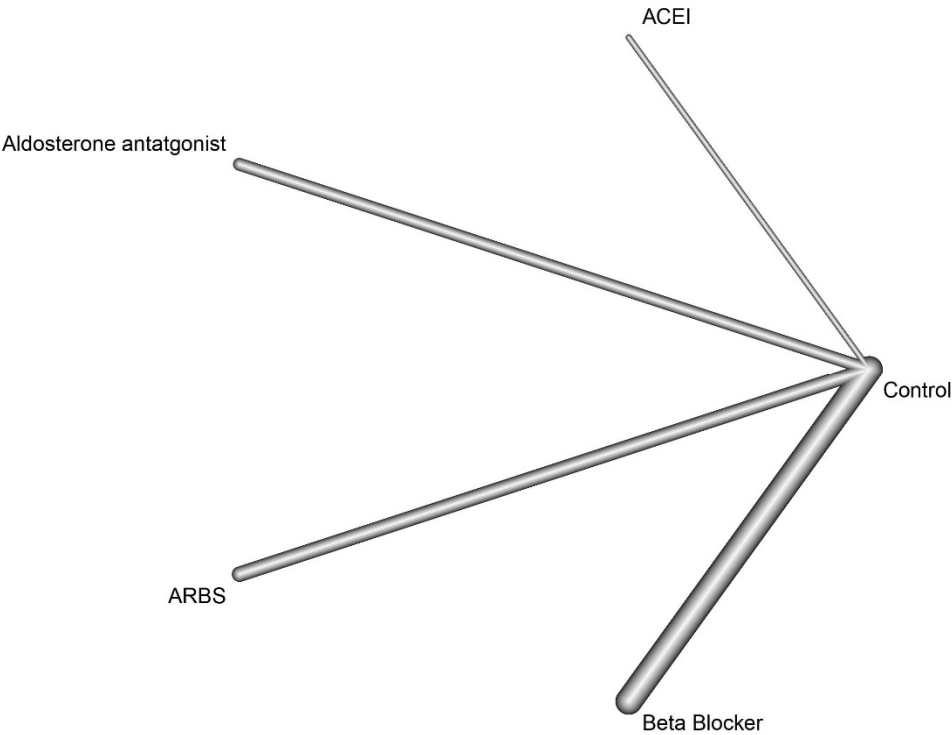

B

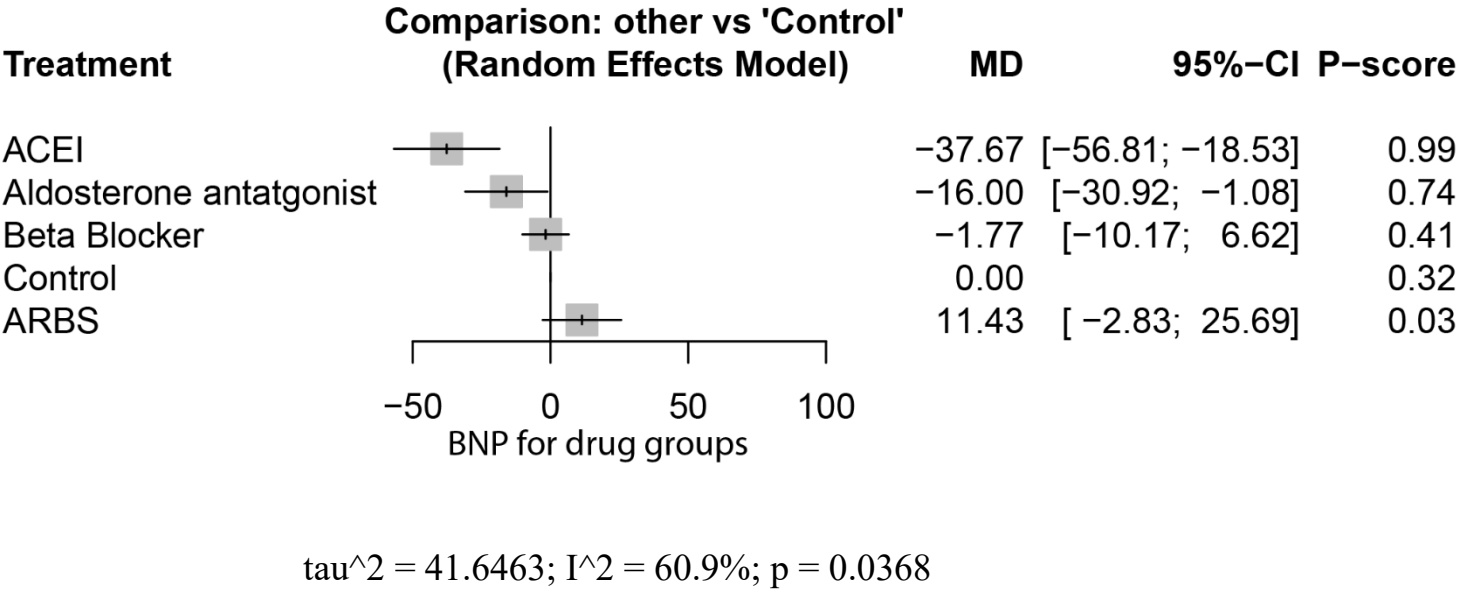

C

| ACEI                    |                        |                       |                       |      |
|-------------------------|------------------------|-----------------------|-----------------------|------|
| -21.67 [-45.94; 2.59]   | Aldosterone antagonist |                       |                       |      |
| -35.90 [-56.80; -15.00] | -14.23 [-31.34; 2.89]  | Beta Blocker          |                       |      |
| -37.67 [-56.81; -18.53] | -16.00 [-30.92; -1.08] | -1.77 [-10.17; 6.62]  | Control               |      |
| -49.10 [-72.97; -25.23] | -27.43 [-48.06; -6.80] | -13.20 [-29.75; 3.34] | -11.43 [-25.69; 2.83] | ARBS |

Supplement: Supplementary file 4 — Additional file 4: Supplementary Figure 4. BNP (A) Network graph revealing direct evidence between the assessed drug families. (B) A forest plot comparing all drug families with control. (C) The league table represents the network meta-analysis estimates for all drug families' comparisons. [file 40959_2023_159_MOESM4_ESM.pdf]

A

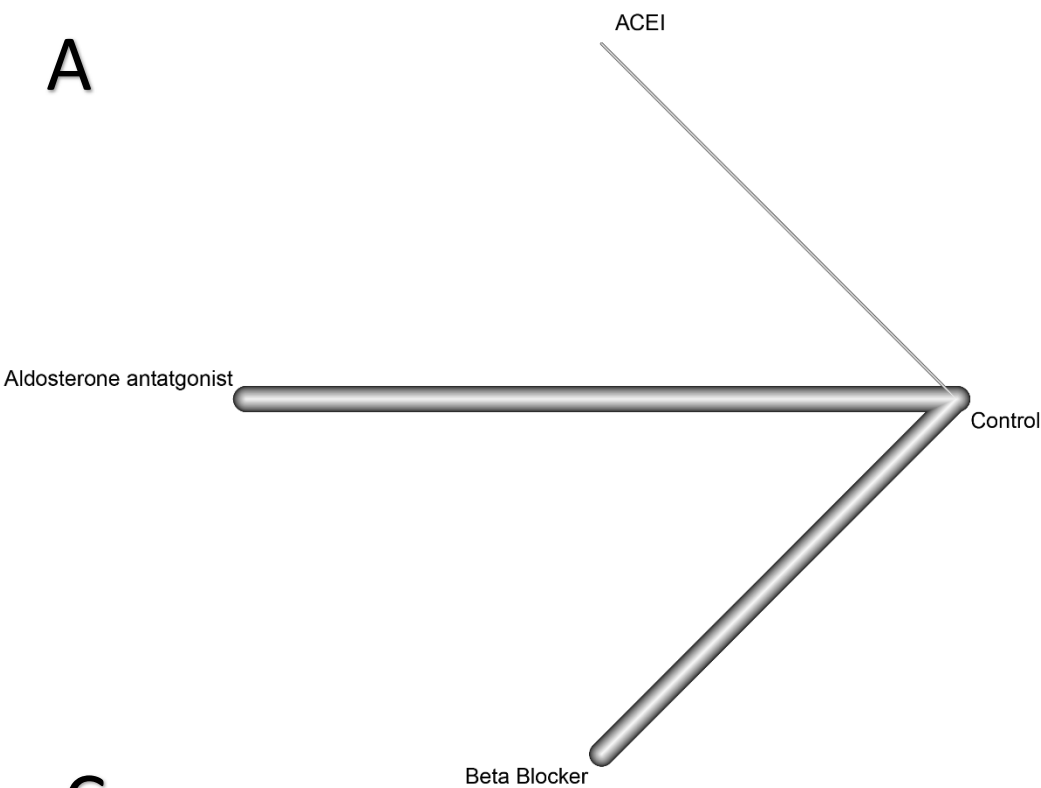

B

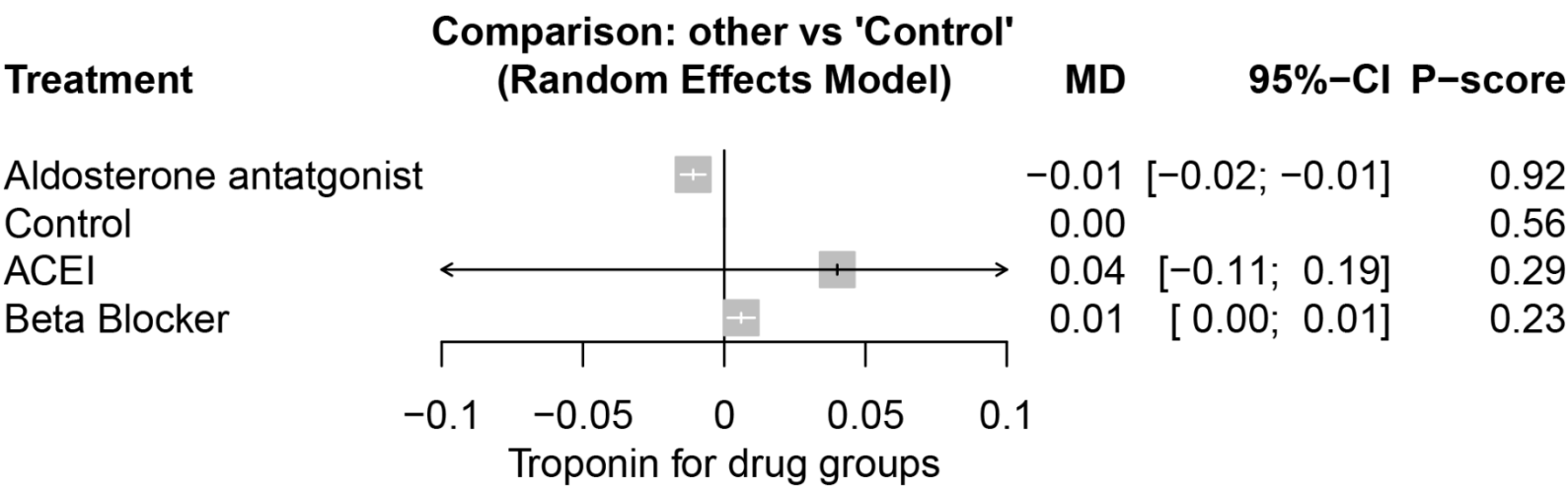

C

|                         |                     |                    |              |
|-------------------------|---------------------|--------------------|--------------|
| Aldosterone antatgonist |                     |                    |              |
| -0.01 [-0.02; -0.01]    | Control             |                    |              |
| -0.05 [-0.20; 0.10]     | -0.04 [-0.19; 0.11] | ACEI               |              |
| -0.02 [-0.02; -0.01]    | -0.01 [-0.01; 0.00] | 0.03 [-0.12; 0.18] | Beta Blocker |

Supplement: Supplementary file 5 — Additional file 5: Supplementary Figure 5. Troponin (A) Network graph revealing direct evidence between the assessed drug families. (B) A forest plot comparing all drug families with control. (C) The league table represents the network meta-analysis estimates for all drug families' comparisons. [file 40959_2023_159_MOESM5_ESM.pdf]

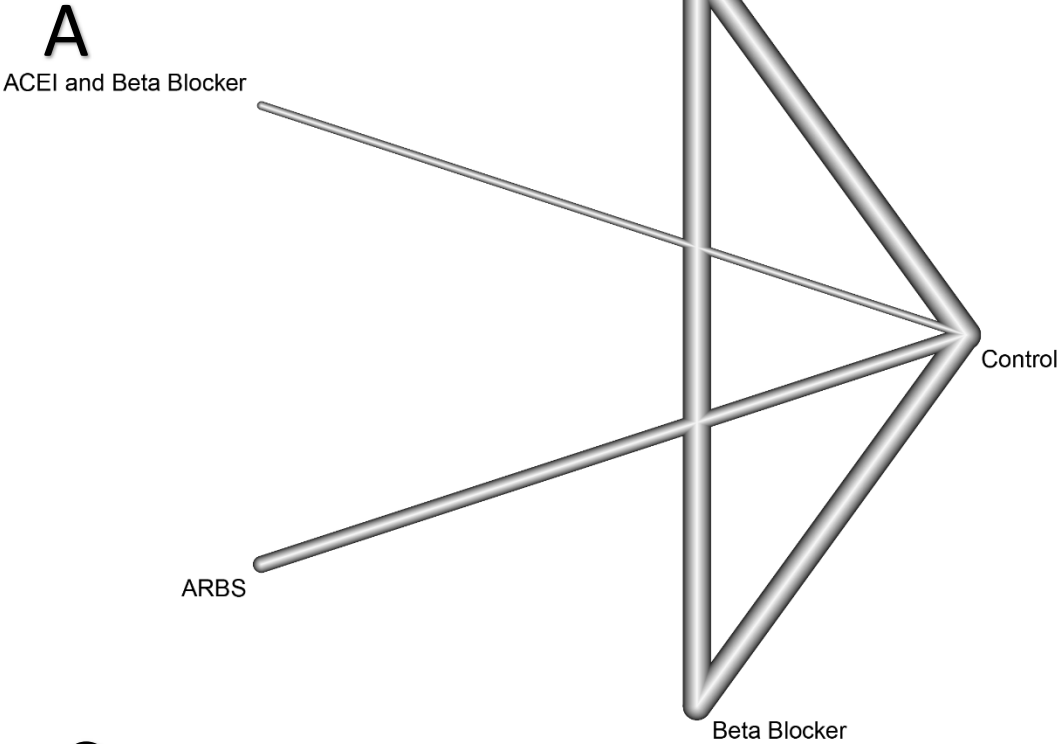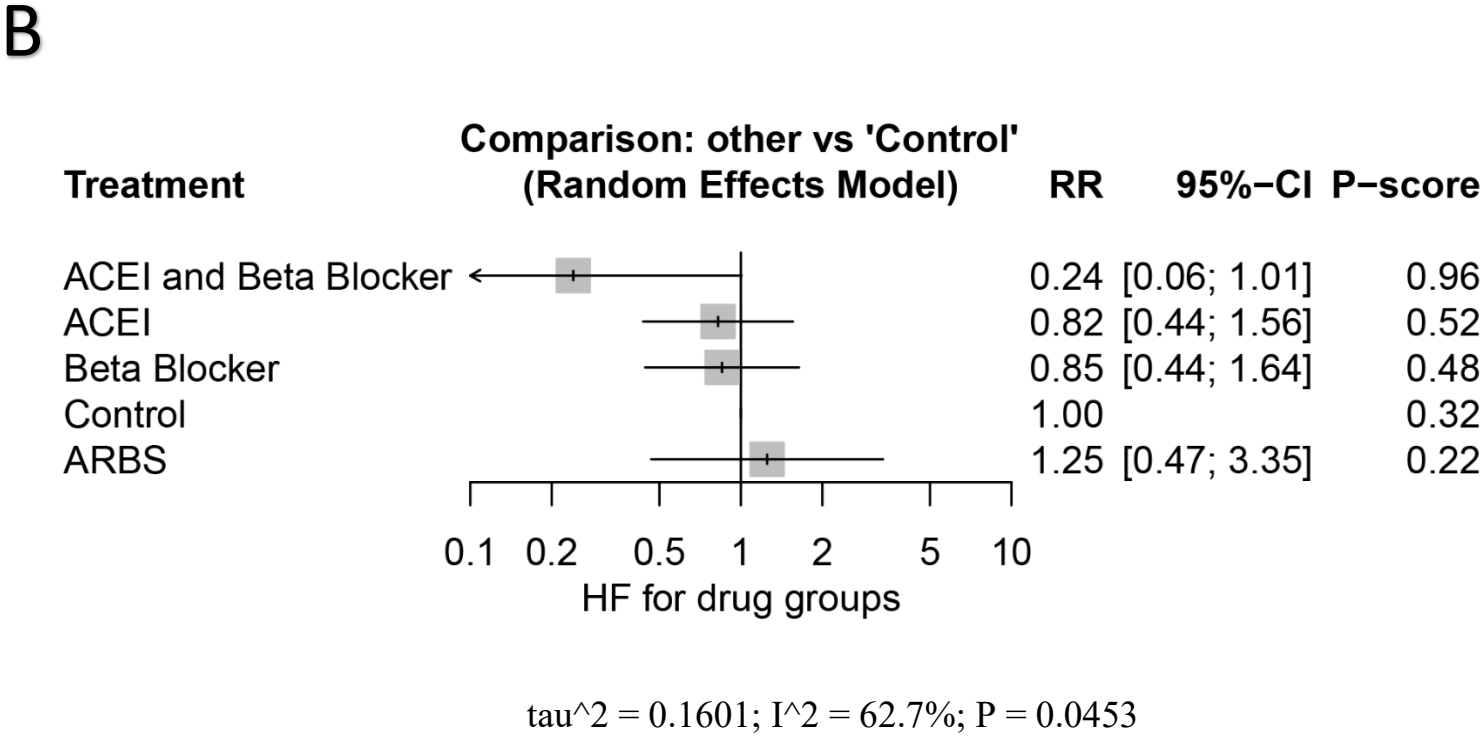

**C**

| ACEI and Beta Blocker |                   |                   |                   |      |
|-----------------------|-------------------|-------------------|-------------------|------|
| 0.29 [0.06; 1.40]     | ACEI              |                   |                   |      |
| 0.28 [0.06; 1.36]     | 0.97 [0.50; 1.86] | Beta Blocker      |                   |      |
| 0.24 [0.06; 1.01]     | 0.82 [0.44; 1.56] | 0.85 [0.44; 1.64] | Control           |      |
| 0.19 [0.03; 1.09]     | 0.66 [0.20; 2.13] | 0.68 [0.21; 2.23] | 0.80 [0.30; 2.14] | ARBS |

Supplement: Supplementary file 6 — Additional file 6: Supplementary Figure 6. Heart Failure (A) Network graph revealing direct evidence between the assessed drug families. (B) A forest plot comparing all drug families with control. (C) The league table represents the network meta-analysis estimates for all drug families' comparisons. [file 40959_2023_159_MOESM6_ESM.pdf]
