# Supplementary material for: Efficacy and safety of cardioprotective drugs in chemotherapy-induced cardiotoxicity: an updated systematic review & network meta-analysis
Source: Cardiooncology. 2023 Feb 18;9:10. doi: 10.1186/s40959-023-00159-0 (PMC9938608; doi:10.1186/s40959-023-00159-0)
Supplement: Supplementary file 2 — Additional file 2: Supplementary Figure 2. LVEF (A) Network graph revealing direct evidence between the assessed drug families. (B) A forest plot comparing all drug families with control. (C) The league table represents the network meta-analysis estimates for all drug families' comparisons. [file 40959_2023_159_MOESM2_ESM.pdf]

A

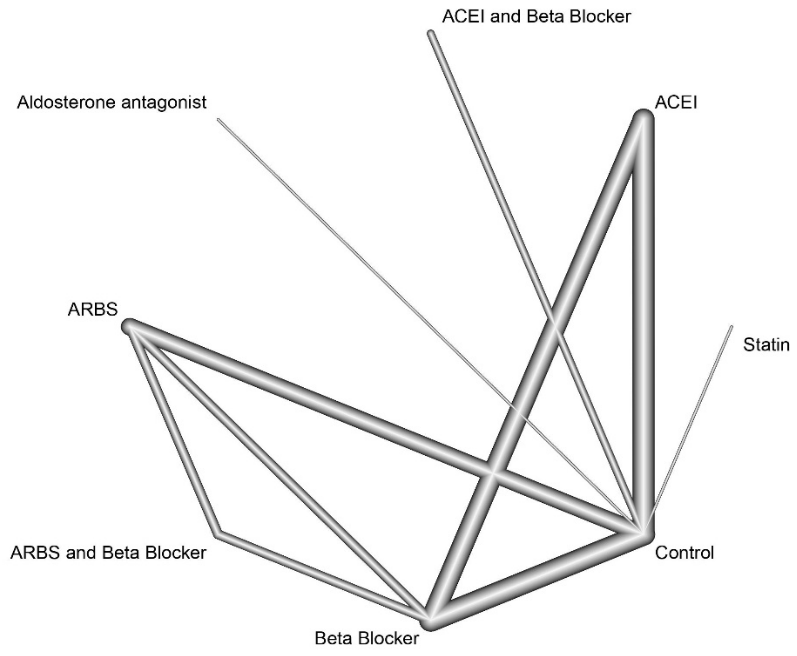

B

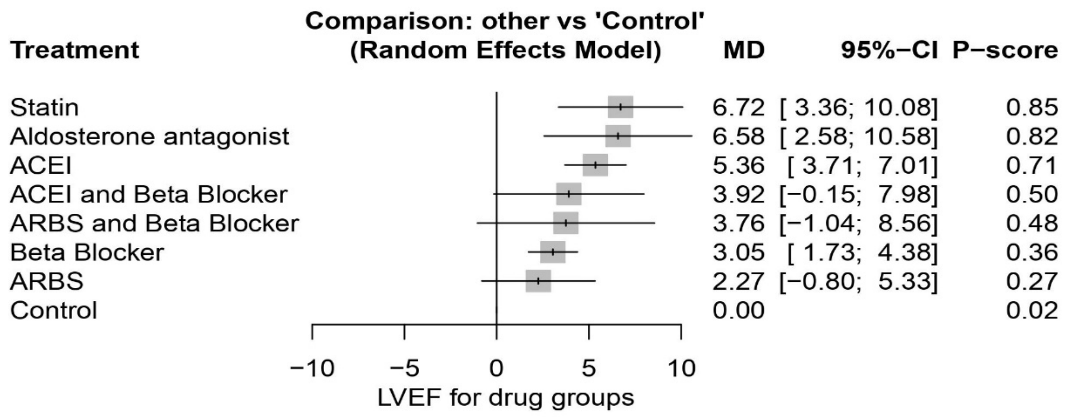

tau-squared = 6.8806; I-squared = 98.3%; P < 0.001

C

**Statin**							
0.14<br>[-5.08; 5.36]	**Aldosterone antagonist**						
1.36<br>[-2.38; 5.11]	1.22<br>[-3.10; 5.55]	**ACEI**					
2.81<br>[-2.47; 8.08]	2.66<br>[-3.04; 8.36]	1.44<br>[-2.94; 5.83]	**ACEI and Beta Blocker**				
2.96<br>[-2.89; 8.82]	2.82<br>[-3.42; 9.06]	1.60<br>[-3.41; 6.61]	0.16<br>[-6.13; 6.44]	**ARBS and Beta Blocker**			
**3.67**<br>**[0.06; 7.28]**	3.53<br>[-0.68; 7.74]	**2.30**<br>**[0.43; 4.18]**	0.86<br>[-3.41; 5.14]	0.71<br>[-4.03; 5.44]	**Beta Blocker**		
4.46<br>[-0.09; 9.01]	4.31<br>[-0.72; 9.35]	3.09<br>[-0.34; 6.53]	1.65<br>[-3.44; 6.74]	1.49<br>[-3.24; 6.23]	0.79<br>[-2.37; 3.94]	**ARBS**	
**6.72**<br>**[3.36; 10.08]**	**6.58**<br>**[2.58; 10.58]**	**5.36**<br>**[3.71; 7.01]**	3.92<br>[-0.15; 7.98]	3.76<br>[-1.04; 8.56]	**3.05**<br>**[1.73; 4.38]**	2.27<br>[-0.80; 5.33]	**Control**
