# Supplementary material for: Efficacy and safety of cardioprotective drugs in chemotherapy-induced cardiotoxicity: an updated systematic review & network meta-analysis
Source: Cardiooncology. 2023 Feb 18;9:10. doi: 10.1186/s40959-023-00159-0 (PMC9938608; doi:10.1186/s40959-023-00159-0)
Supplement: Supplementary file 7 — Additional file 7: Supplementary Table 1. Search strategy. [file 40959_2023_159_MOESM7_ESM.docx]

**Table 3:** search strategy

***********************************************************

| **SEARCH STRATEGY IN GENERAL:**  (Cancer* OR carcinoma* OR neoplasm* OR tumor OR tumour* OR malignan* OR “Antineoplastic Agent*” OR chemotherap* OR “antineoplastic drug*” OR “anticancer agent*” OR anticancer drug* OR anthracyclines OR daunorubicin OR doxorubicin OR epirubicin OR Aclarubicin OR carubicin OR idarubicin OR nogalamycin OR plicamycin OR Trastuzumab OR herceptin)  AND  (Cardiotoxic* OR cardioprotect* OR cardiomyopath* OR “myocardial Disease*” OR “cardiac dysfunction”)  AND  (Statin* OR “Hydroxymethylglutaryl-CoA Reductase Inhibitors” OR “Hydroxymethylglutaryl CoA Reductase Inhibitors” OR “hmg coa reductase” OR “hydroxymethylglutaryl-coa reductase” OR atorvastatin OR lovastatin OR pravastatin OR pitavastatin OR rosuvastatin OR fluvastatin OR simvastatin OR “beta blocker*” OR “beta-blocker*” OR “beta-adrenoceptor block*” OR atenolol OR propranolol OR metoprolol OR arotinolol OR betaxolol OR bevantolol OR bisoprolol OR carteolol OR carvedilol OR celiprolol OR sotalol OR nebivolol OR labetalol OR esmolol OR “Angiotensin Converting Enzyme Inhibitor*” OR ACEI OR captopril OR enalapril OR fosinopril OR lisinopril OR perindopril OR ramipril OR quinapril OR benazepril OR cilazapril OR trandolapril OR spirapril OR delapril OR moexipril OR zofenopril OR imidapril OR “Angiotensin II Type 1 Receptor Blocker” OR “Angiotensin II Receptor Blockers” OR “Angiotensin Receptor Blockers” OR ARB OR candesartan OR eprosartan OR irbesartan OR losartan OR olmesartan OR telmisartan OR valsartan OR “aldosterone antagonist*” OR “Aldosterone Receptor Antagonist*” OR “Mineralocorticoid Antagonists” OR spironolactone OR eplerenone OR canrenoate OR Finerenone OR Mexrenone) |
| --- |
| Part of scopus:  (Statin* OR atorvastatin OR lovastatin OR pravastatin OR pitavastatin OR rosuvastatin OR fluvastatin OR simvastatin OR “beta blocker*” OR “beta-blocker*” OR “beta-adrenoceptor block*” OR atenolol OR propranolol OR metoprolol OR arotinolol OR betaxolol OR bevantolol OR bisoprolol OR carteolol OR carvedilol OR celiprolol OR sotalol OR nebivolol OR labetalol OR esmolol OR ACEI OR captopril OR enalapril OR fosinopril OR lisinopril OR perindopril OR ramipril OR quinapril OR benazepril OR cilazapril OR trandolapril OR spirapril OR delapril OR moexipril OR zofenopril OR imidapril OR ARB OR candesartan OR eprosartan OR irbesartan OR losartan OR olmesartan OR telmisartan OR valsartan OR “aldosterone antagonist*” OR “Aldosterone Receptor Antagonist*” OR “Mineralocorticoid Antagonists” OR spironolactone OR eplerenone OR canrenoate OR Finerenone OR Mexrenone) |
